# Supplementary material for: Evaluating the inhibitory efficacy of Oxalis phytocompounds on monoamine oxidase B: An integrated approach targeting age related neurodegenerative diseases through molecular docking and dynamics simulations
Source: PLoS One. 2025 Jul 30;20(7):e0329168. doi: 10.1371/journal.pone.0329168 (PMC12309996; doi:10.1371/journal.pone.0329168)
Supplement: S1 File — S1 Fig. Binding modes and interactions of ligands with MAO-B: Ribbon model representation (left) showing the orthosteric pocket of MAO-B (PDB ID: 4A79) and 3D interaction profiles (right) illustrating the interacting amino acid residues of the enzyme with the top 8 ligands, along with the native ligand. S2 Fig. RMSD of protein backbone complexed with top 8 ligands relative to protein backbone along with apo protein (black); Complex 1; violet = beta-sitosterol, Complex 2; green = squalene, Complex 3; red = etretinate, Complex 4; cyan = rhoifolin, Complex 5; magenta = swertisin, Complex 6; blue = phloridzin, Complex 7; orange = rhapontin, Complex 8; maroon = diosmetin-7-O-beta-D-glucopyranoside). S3 Fig. RMSF curves of alpha-carbon atoms of protein backbone in top 8 protein-ligand complexes relative to the protein backbone along with that of apo protein (black); Complex 1; violet = beta-sitosterol, Complex 2; green = squalene, Complex 3; red = etretinate, Complex 4; cyan = rhoifolin, Complex 5; magenta = swertisin, Complex 6; blue = phloridzin, Complex 7; orange = rhapontin, Complex 8; maroon = diosmetin-7-O-beta-D-glucopyranoside. S4 Fig. Variation of SASA of proteins in top 8 protein ligand complexes compared to that of the apo protein (black). (Complex 1; violet = beta-sitosterol, Complex 2; green = squalene, Complex 3; red = etretinate, Complex 4; cyan = rhoifolin, Complex 5; magenta = swertisin, Complex 6; blue = phloridzin, Complex 7; orange = rhapontin, Complex 8; maroon = diosmetin-7-O-beta-D-glucopyranoside). S5 Fig. Variation of the radius of gyration of the protein in top 8 protein-ligand complexes and the apo protein (black) obtained from the MDS trajectories; Complex 1 (violet); Complex 2 (green); Complex 3 (red); Complex4 (cyan); Complex 5 (magenta); Complex 6 (blue); Complex 7 (orange); Complex 8 (maroon). S6 Fig. Variation in hydrogen bond count in the top 8 protein ligand complexes (absent in Complex 2) throughout the MDS; (1) Complex 1 (violet); (3) [file pone.0329168.s001.docx]

**Supplementary Information**

**Evaluating the inhibitory efficacy of *Oxalis* phytocompounds on monoamine oxidase B: An integrated approach targeting age related neurodegenerative diseases through molecular docking and dynamics simulations**

Ram Lal (Swagat) Shrestha^1,2,4¶^, Shiva M.C.^1,2¶^, Ashika Tamang^1,2^, Manila Poudel^2,3^, Nirmal Parajuli^1,2^, Aakar Shrestha^2^, Timila Shrestha^1,2^, Samjhana Bharati^1,2^, Binita Maharjan^1,2^, Bishnu P. Marasini^2,4,5&*^_,_ Jhashanath Adhikari Subin^2,6,&*^

^1^Department of Chemistry, Amrit Campus, Tribhuvan University, Lainchaur, Kathmandu 44600, Nepal

^2^Kathmandu Valley College, Syuchatar Bridge, Kalanki, Kathmandu 44600, Nepal

^3^Department of Biotechnology, National College, Lainchaur, Kathmandu 44600, Nepal

^4^Institute of Natural Resources Innovation, Kalimati, Kathmandu 44600, Nepal

^5^Nepal Health Research Council, Ministry of Health and Population, Ramshah Path, Kathmandu 44600, Nepal

^6^Bioinformatics and Cheminformatics Division, Scientific Research and Training Nepal P. Ltd., Bhaktapur 44800, Nepal

*Corresponding authors

Email address: [subinadhikari2018@gmail.com](mailto:subinadhikari2018@gmail.com),[bishnu.marasini@gmail.com](mailto:bishnu.marasini@gmail.com)

^¶^These authors contributed equally to this work.

^&^These authors also contributed equally to this work.

The supplementary information includes 8 figures and 3 tables.


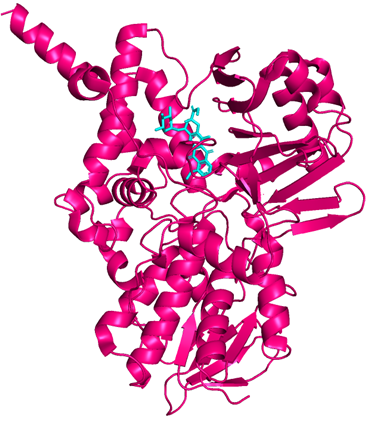

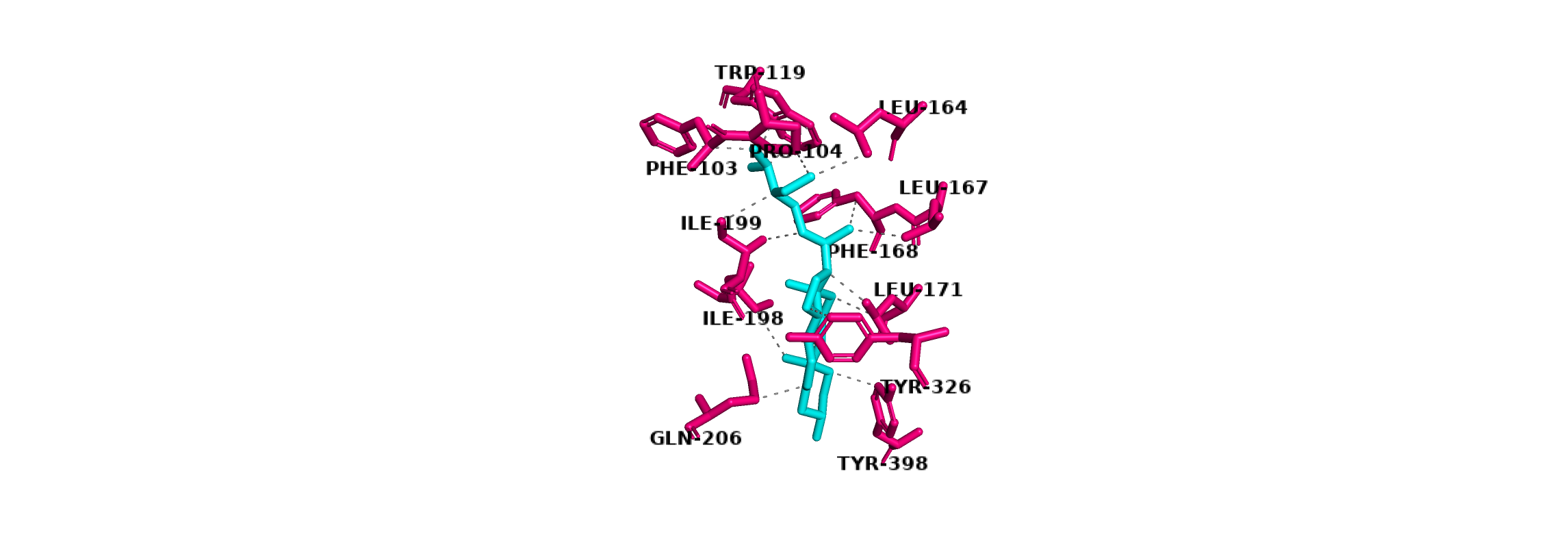


**Beta-sitosterol-MAO-B Complex (1)**


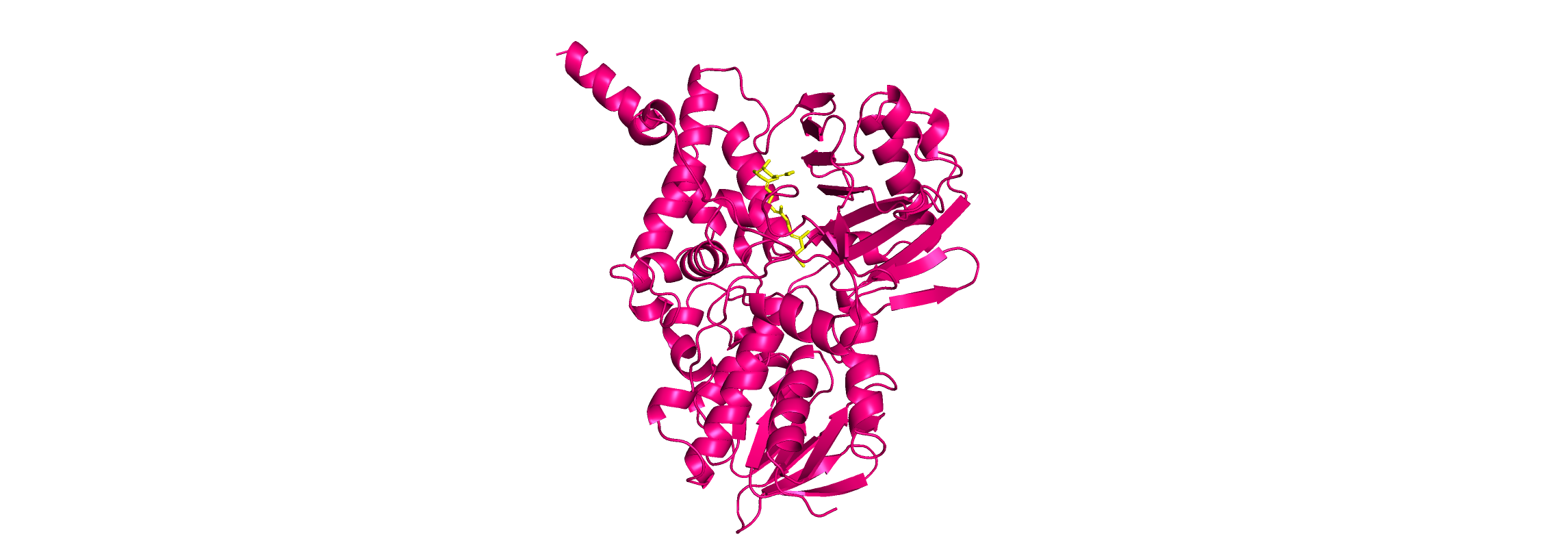

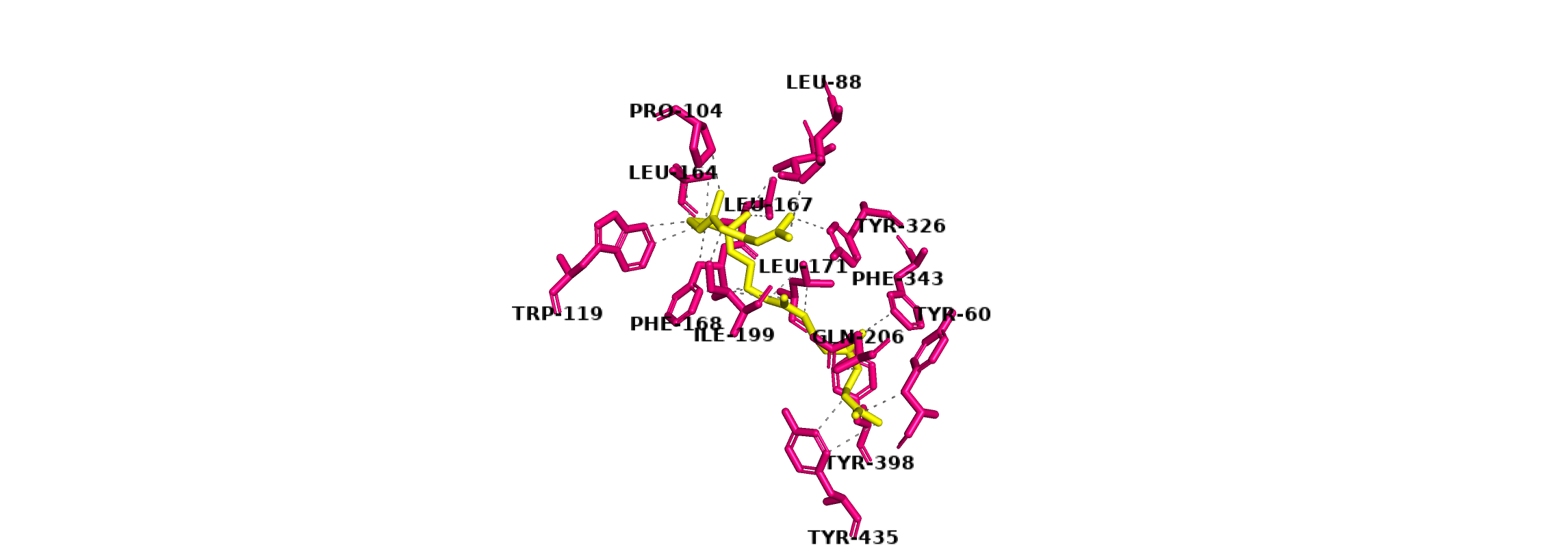


**Squalene-MAO-B Complex (2)**


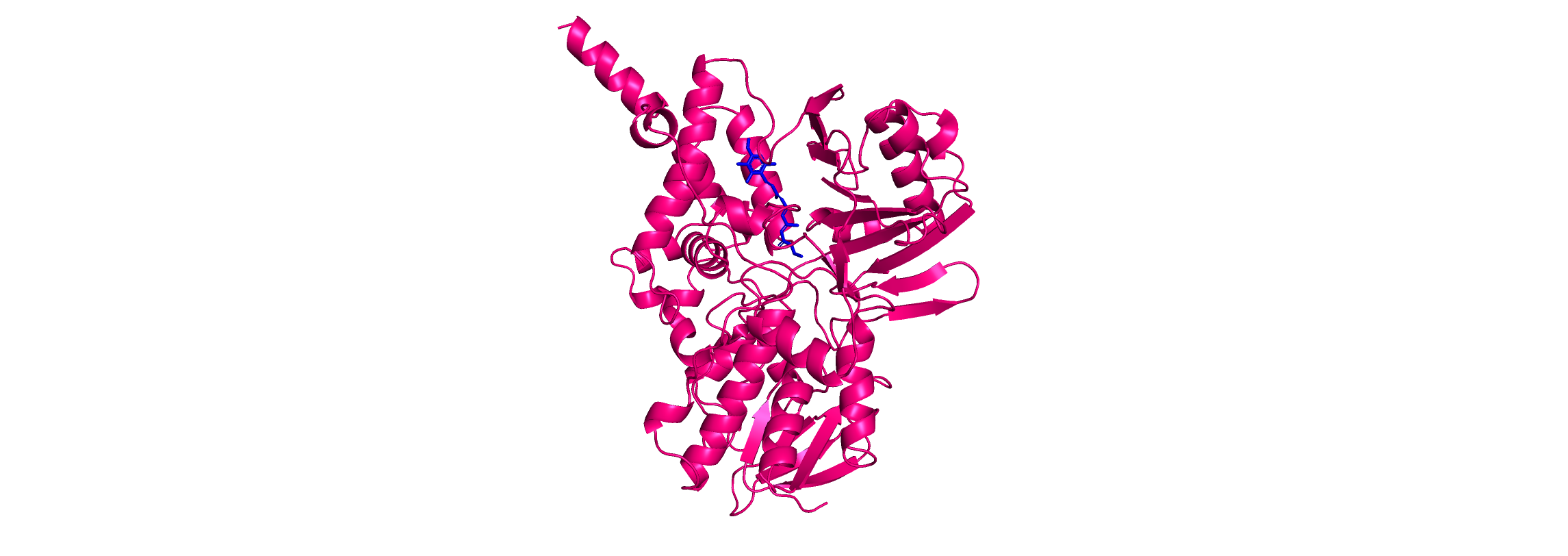

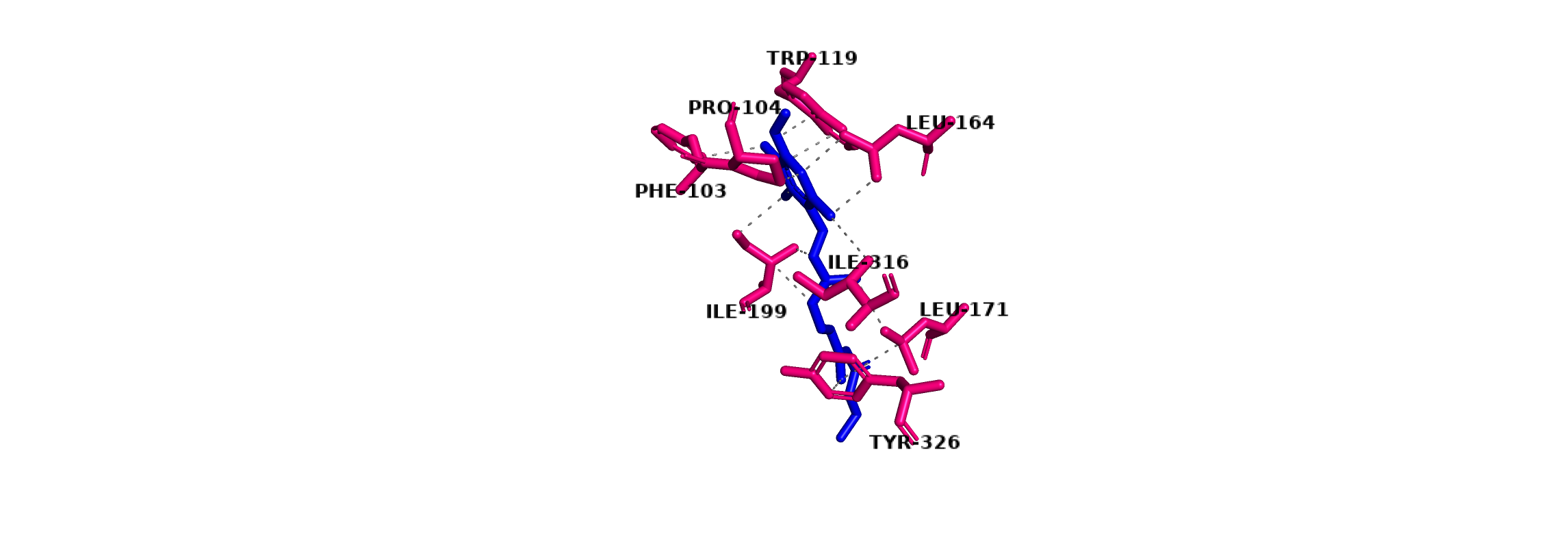


**Etretinate-MAO-B Complex (3)**


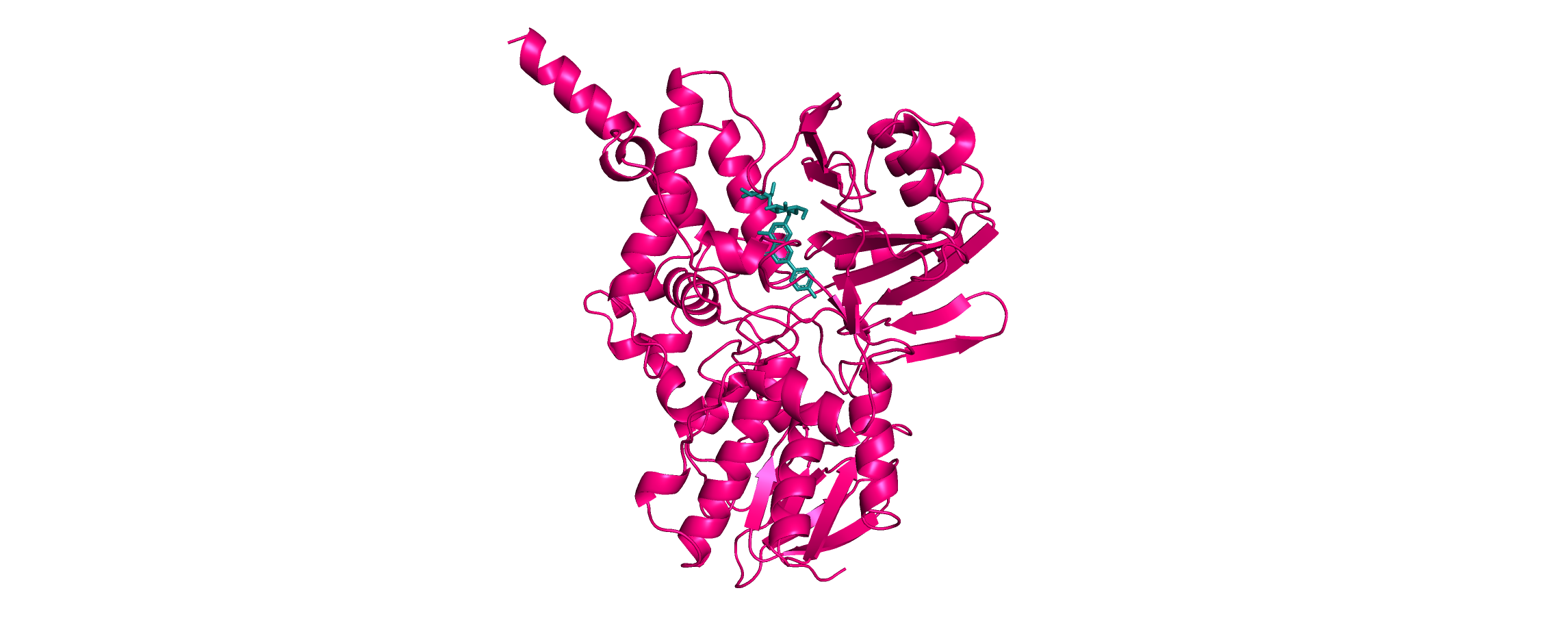

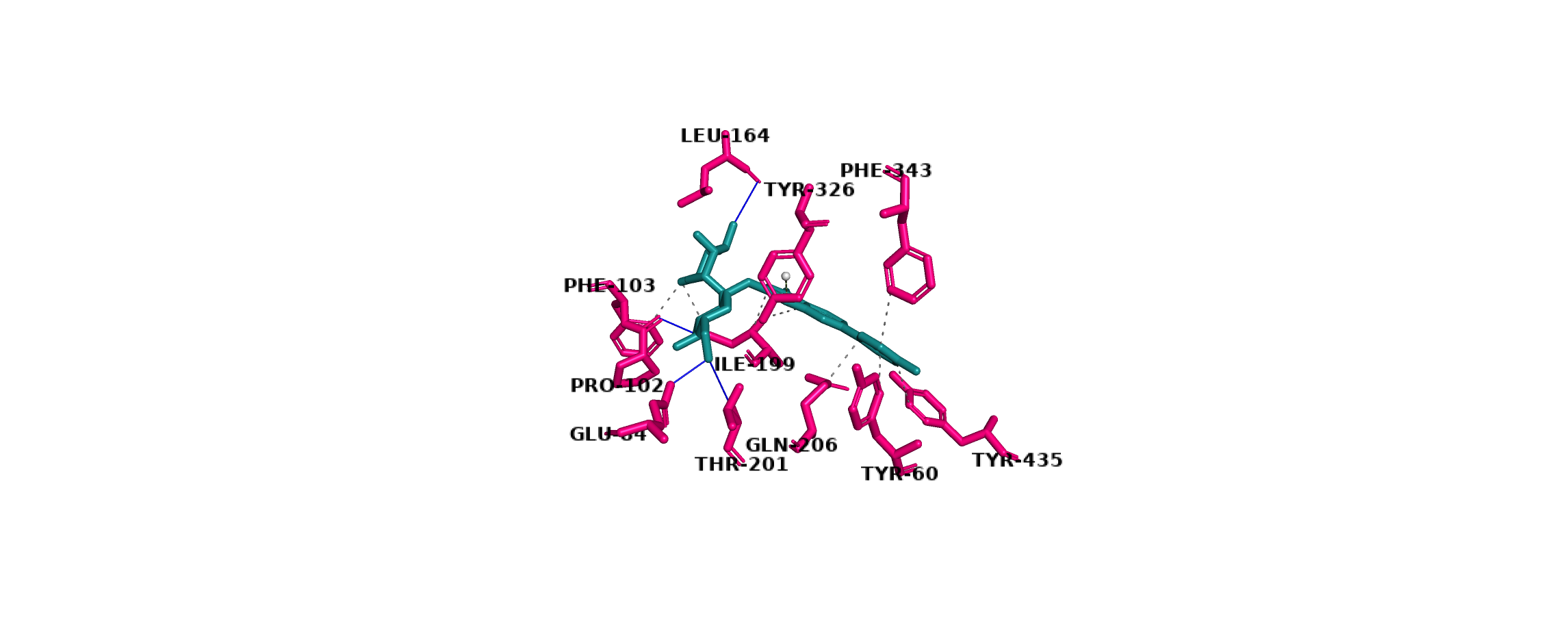


**Rhoifolin-MAO-B Complex (4)**


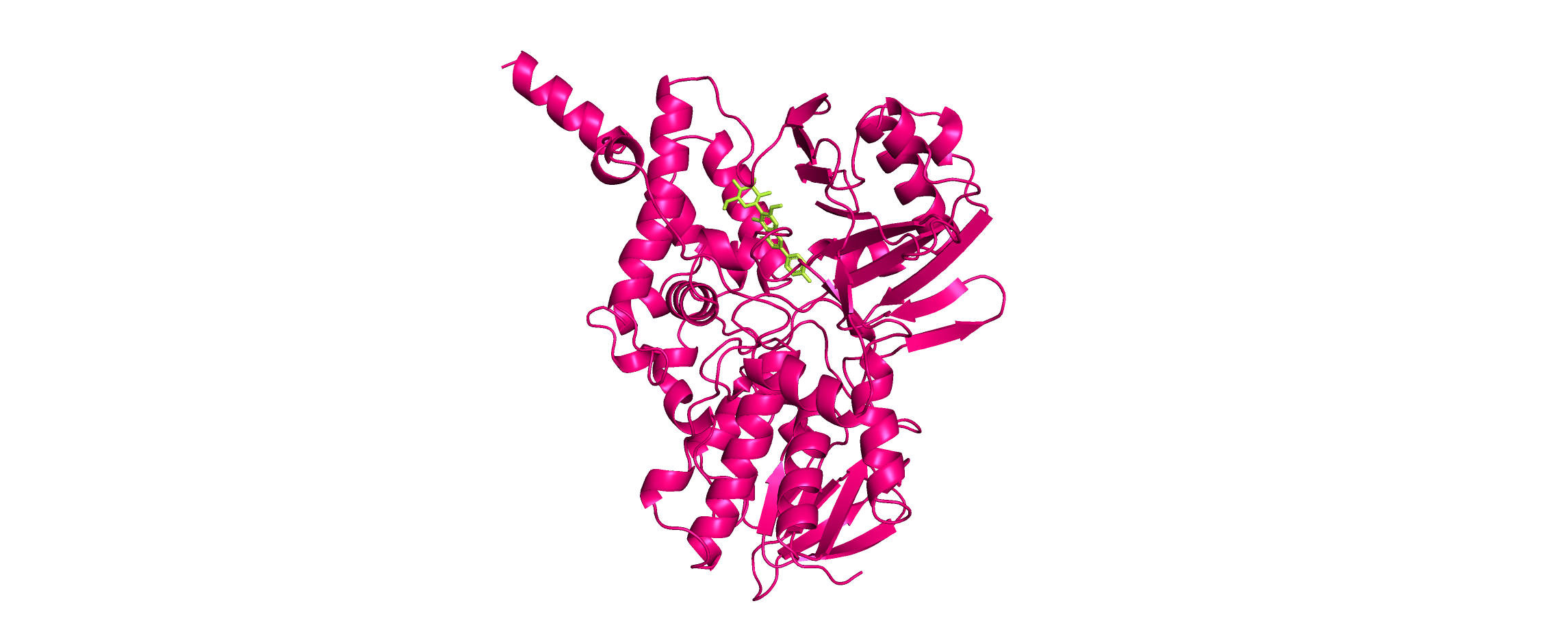

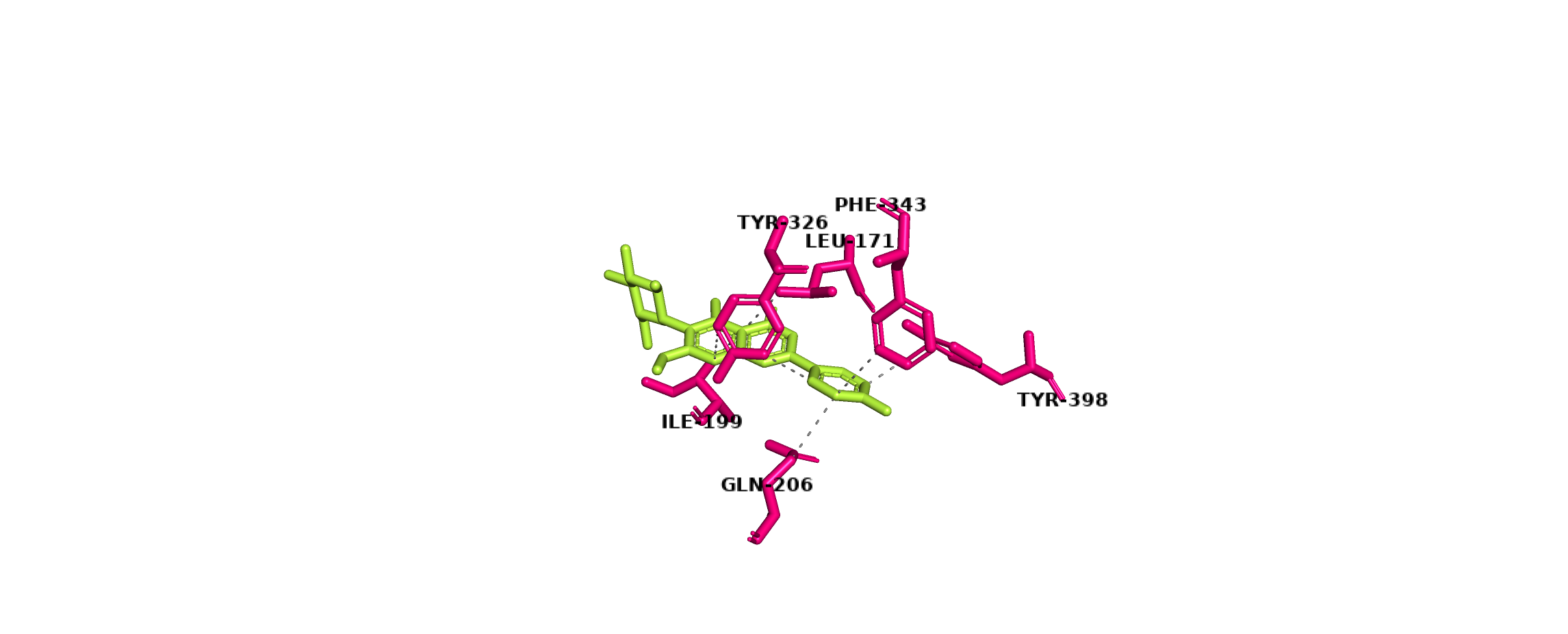


**Swertisin-MAO-B Complex (5)**


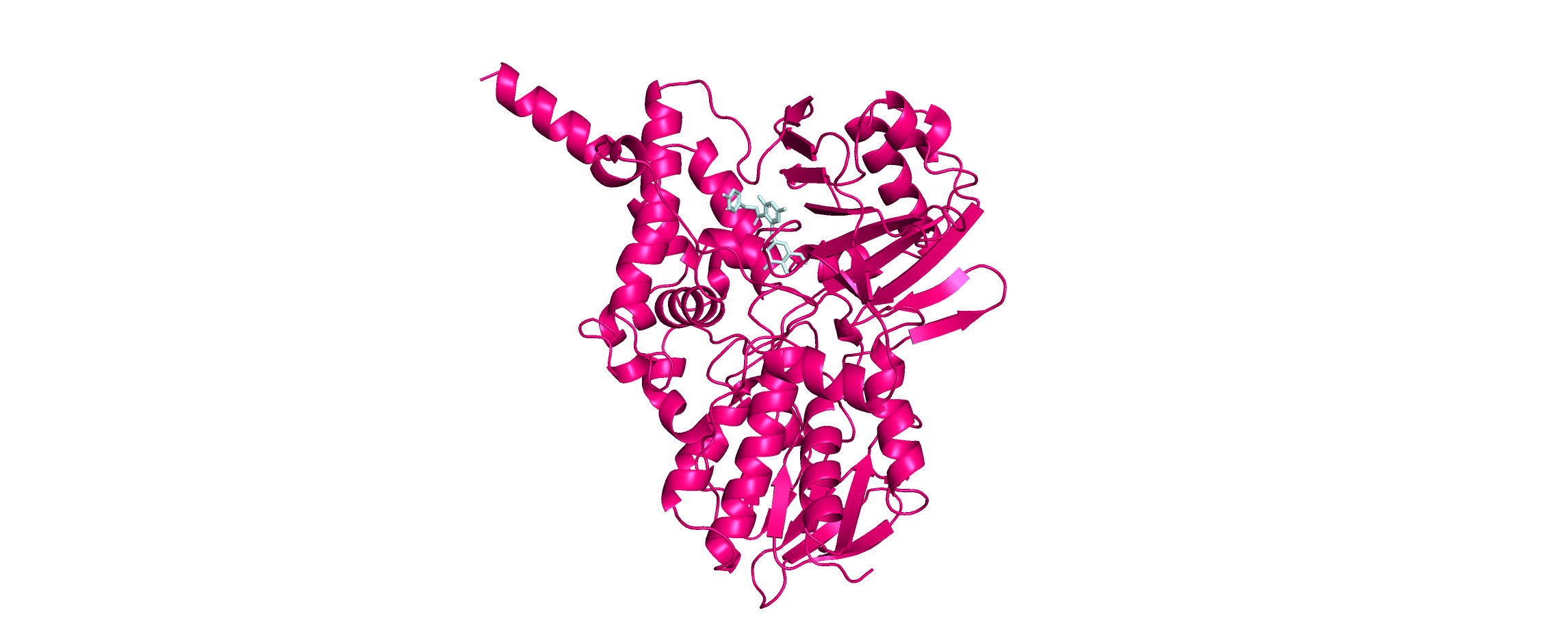

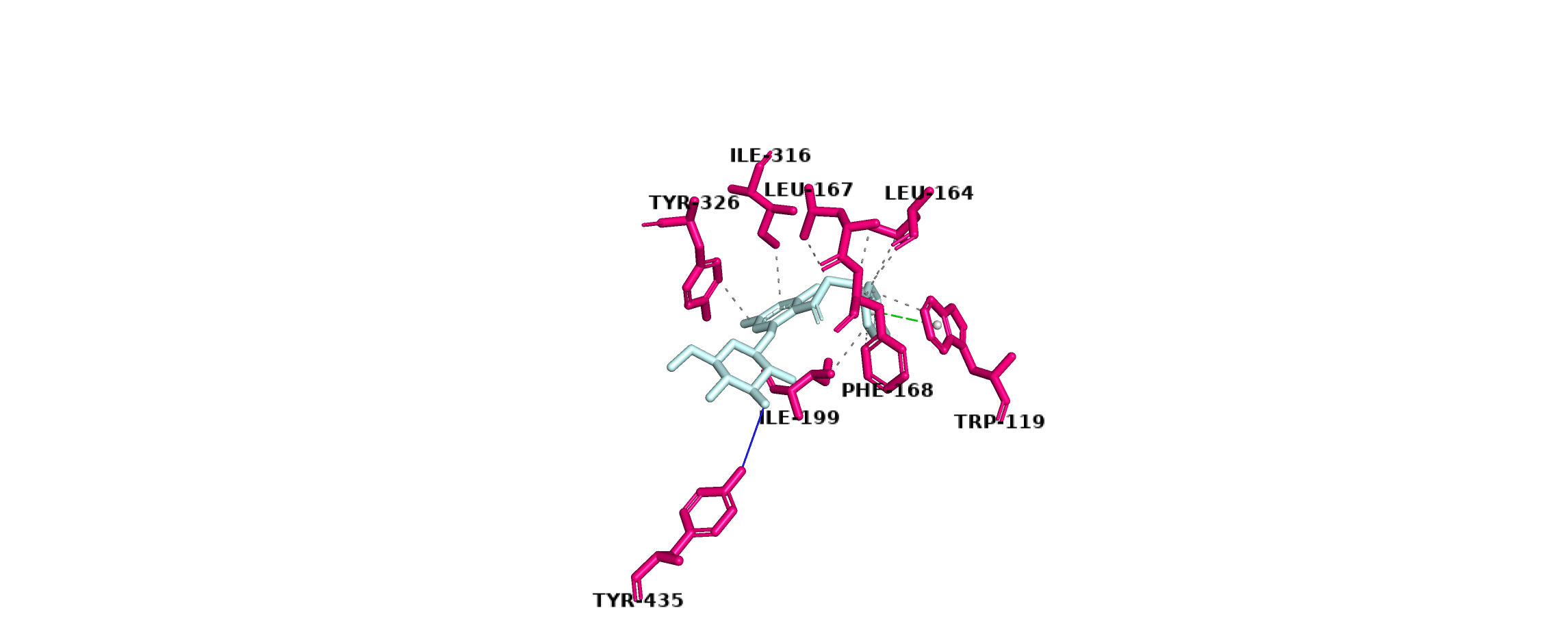


**Phloridzin-MAO-B Complex (6)**


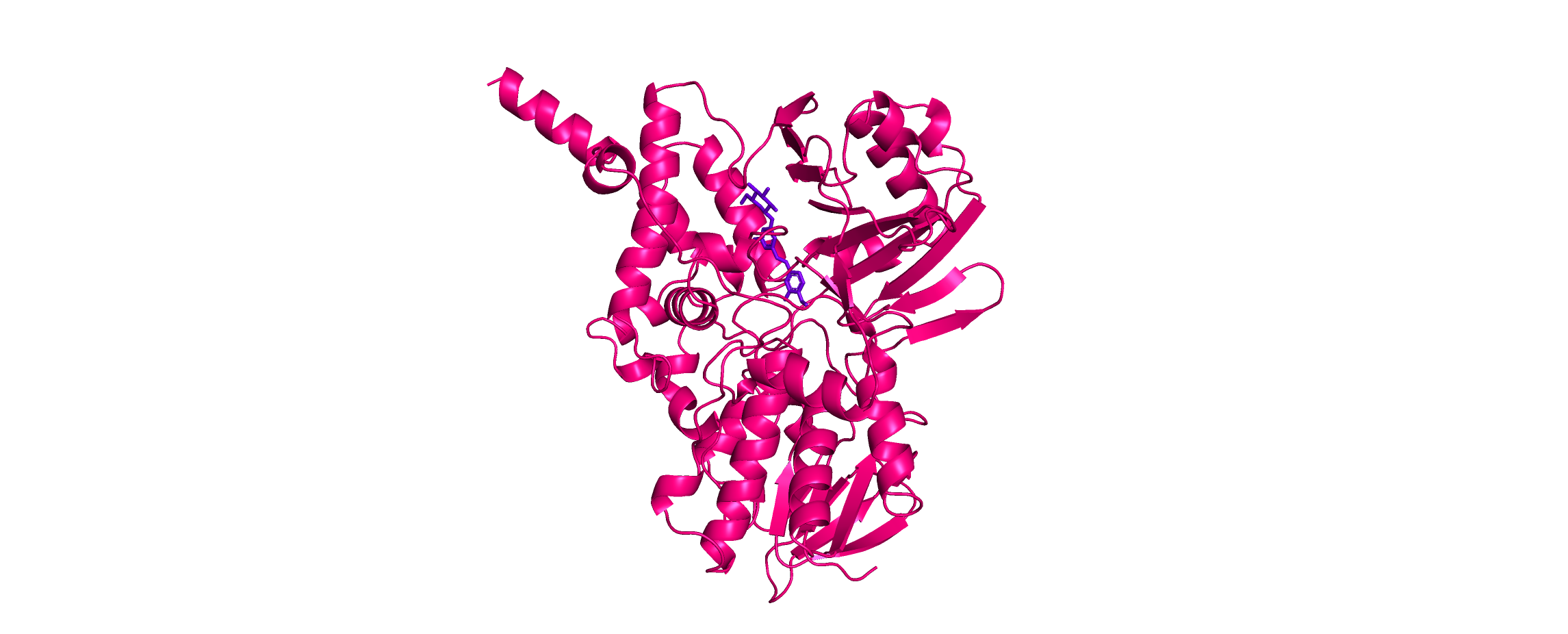

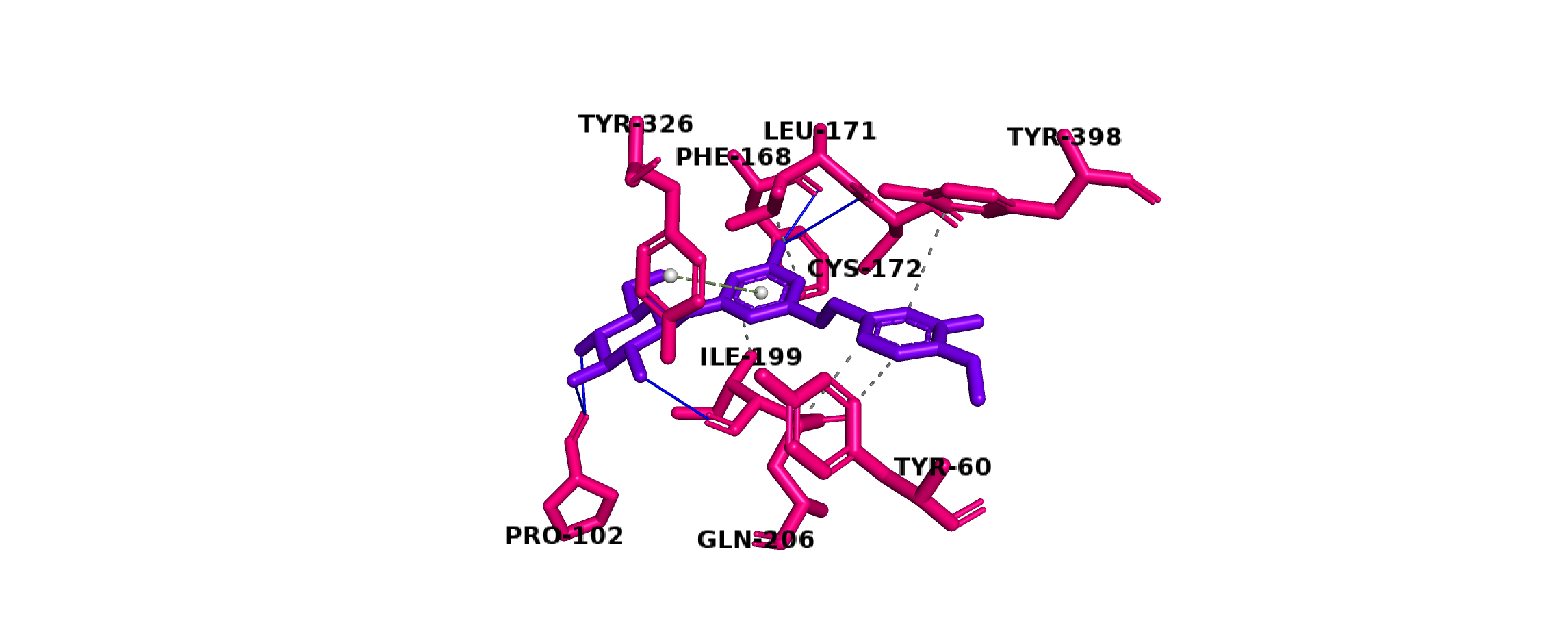


**Rhapontin-MAO-B Complex (7)**


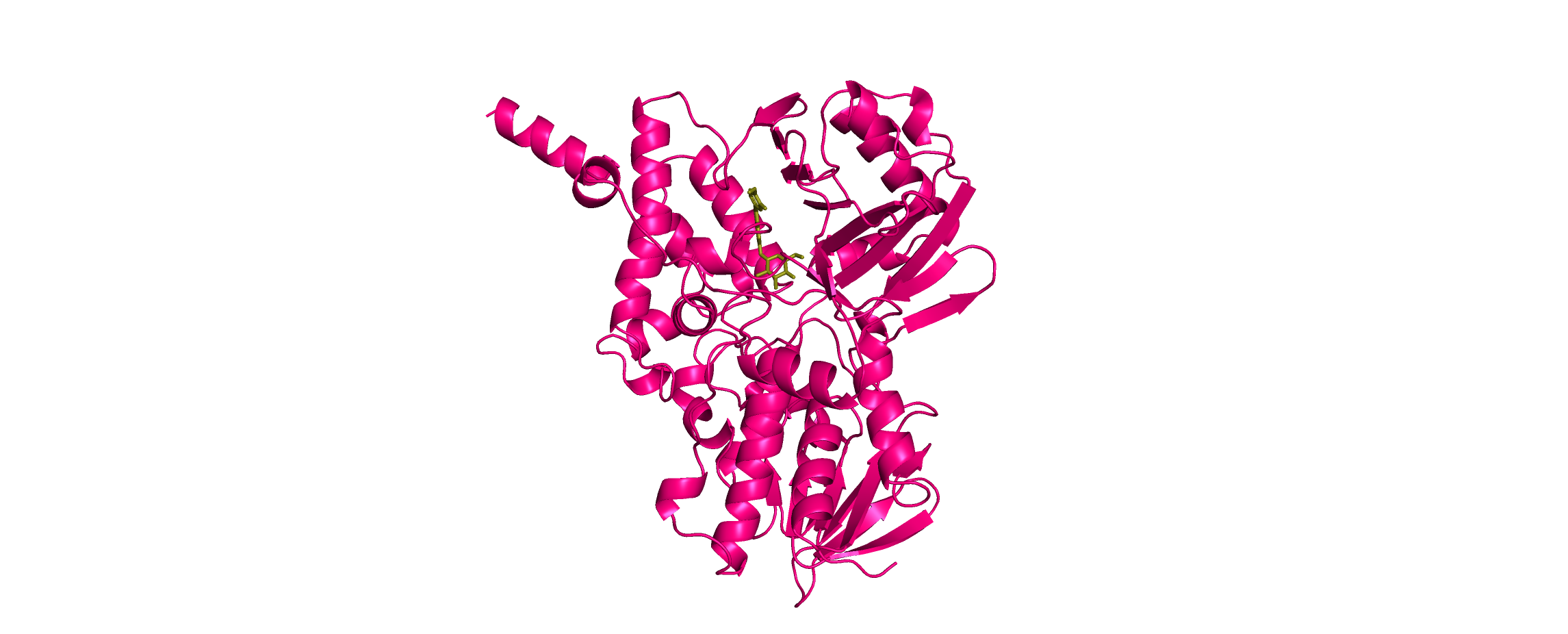

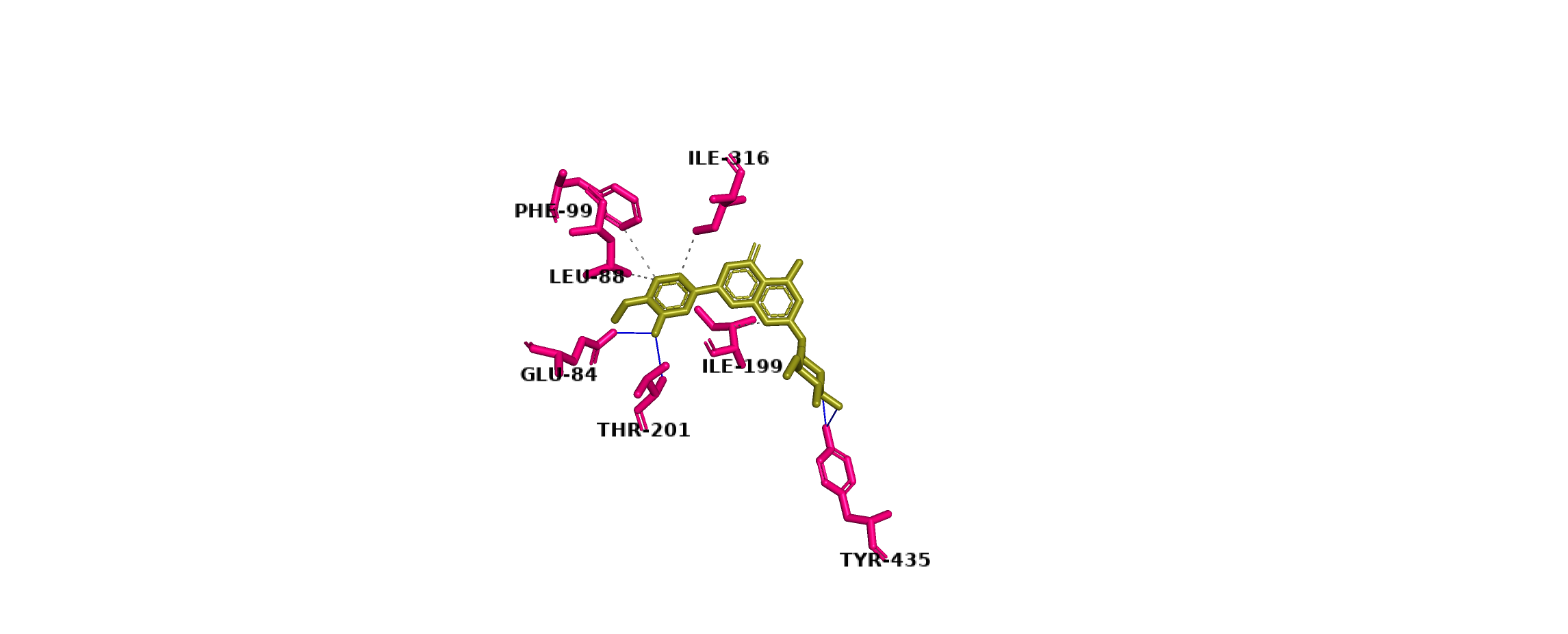


**Diosmetin 7-O-beta-D-glucopyranoside -MAO-B Complex (8)**


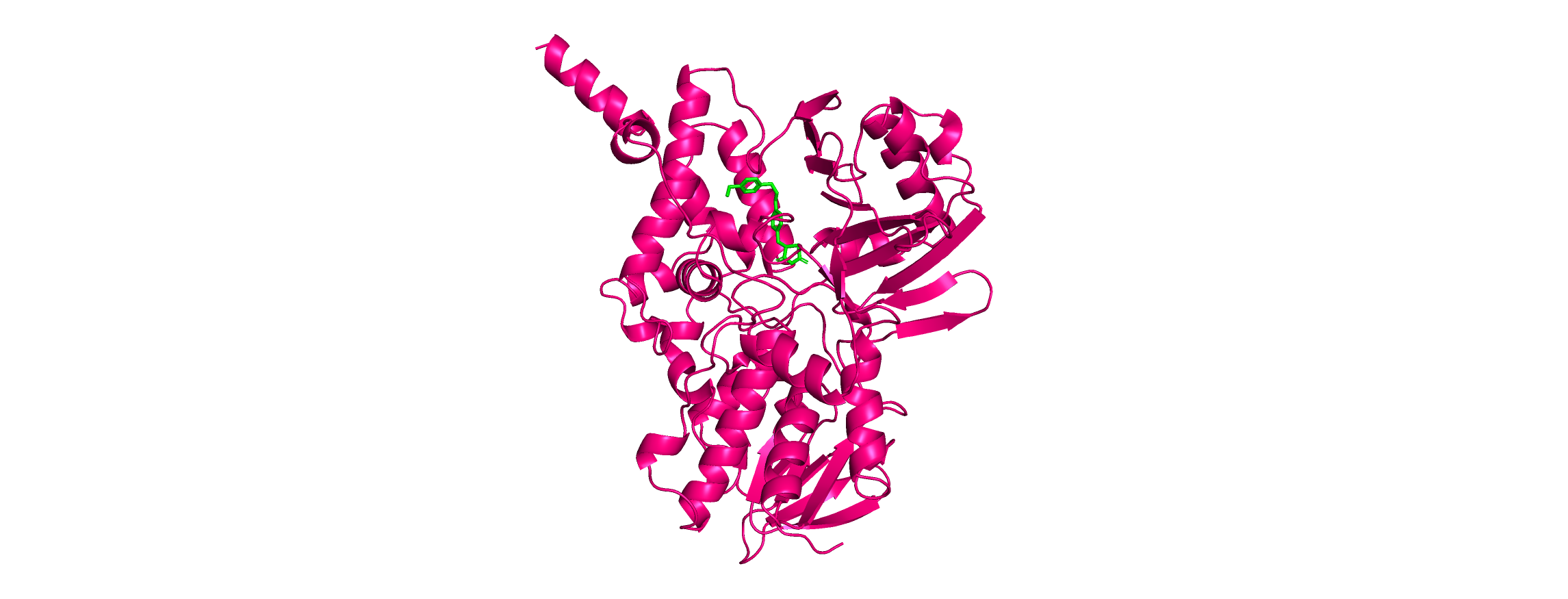

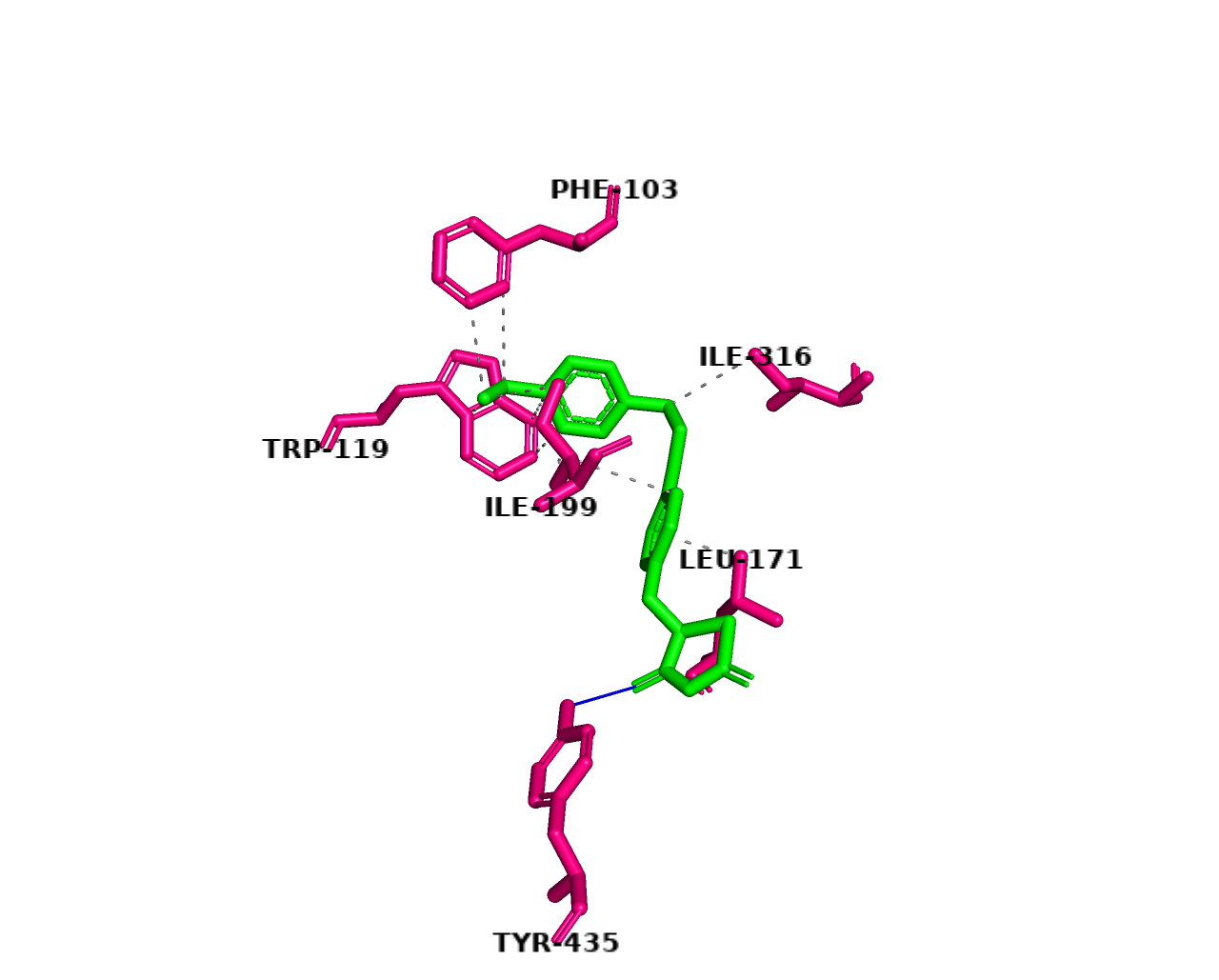


**Native ligand, pioglitazone-MAO-B Complex**


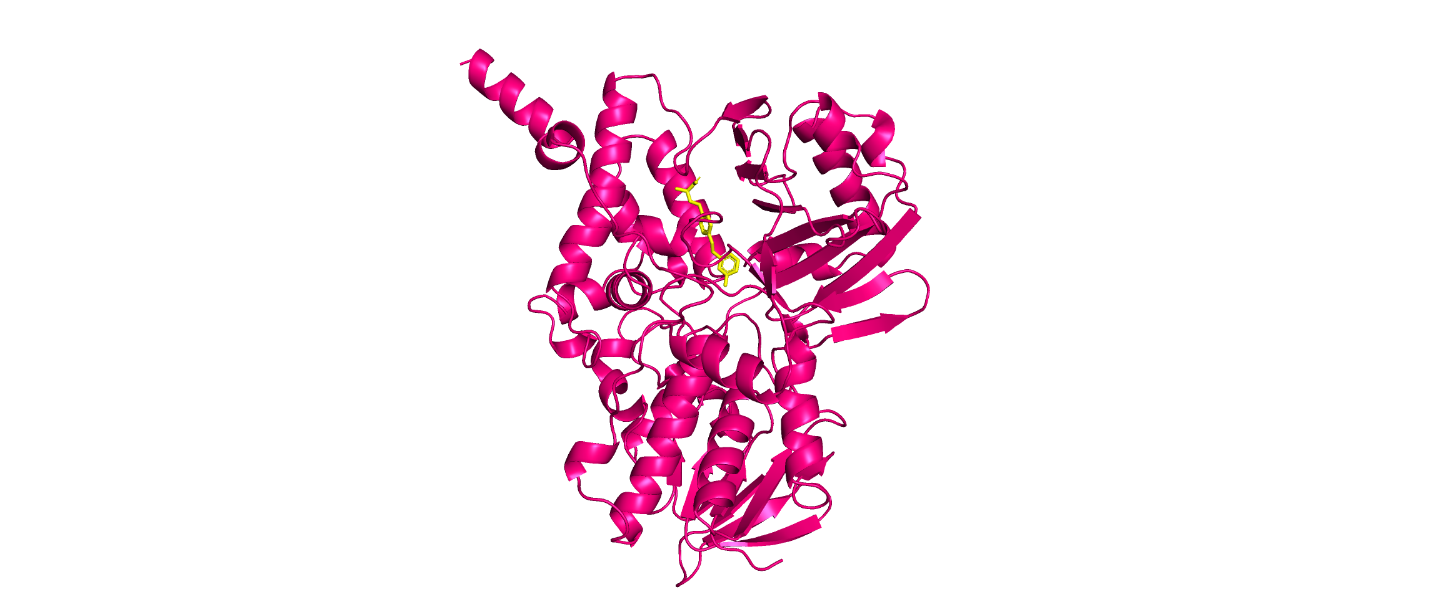

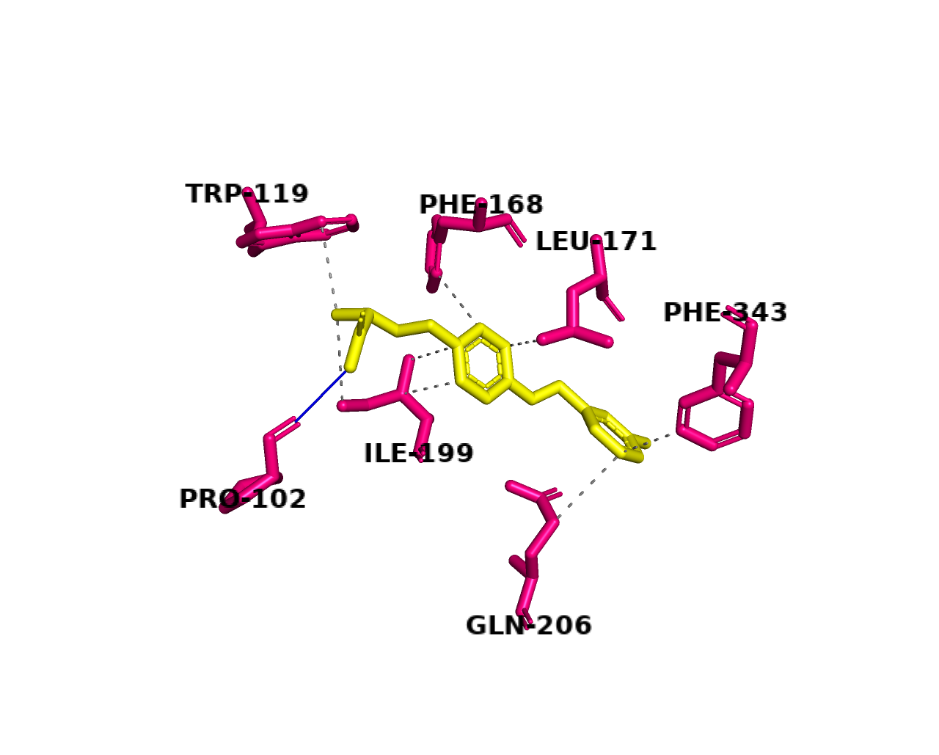


**Safinamide-MAO-B Complex**


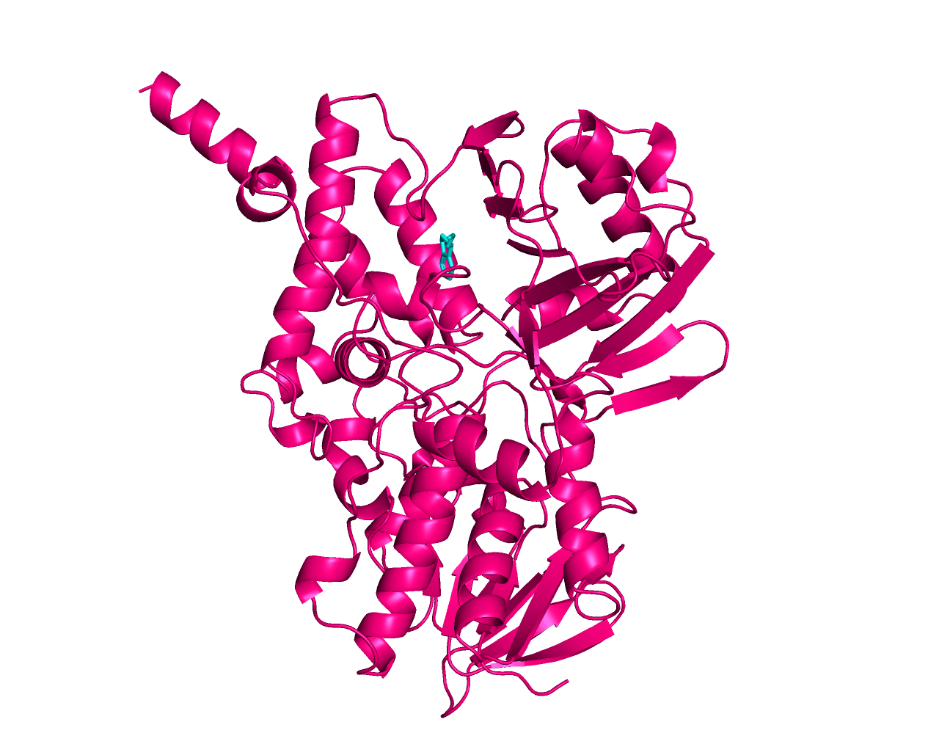

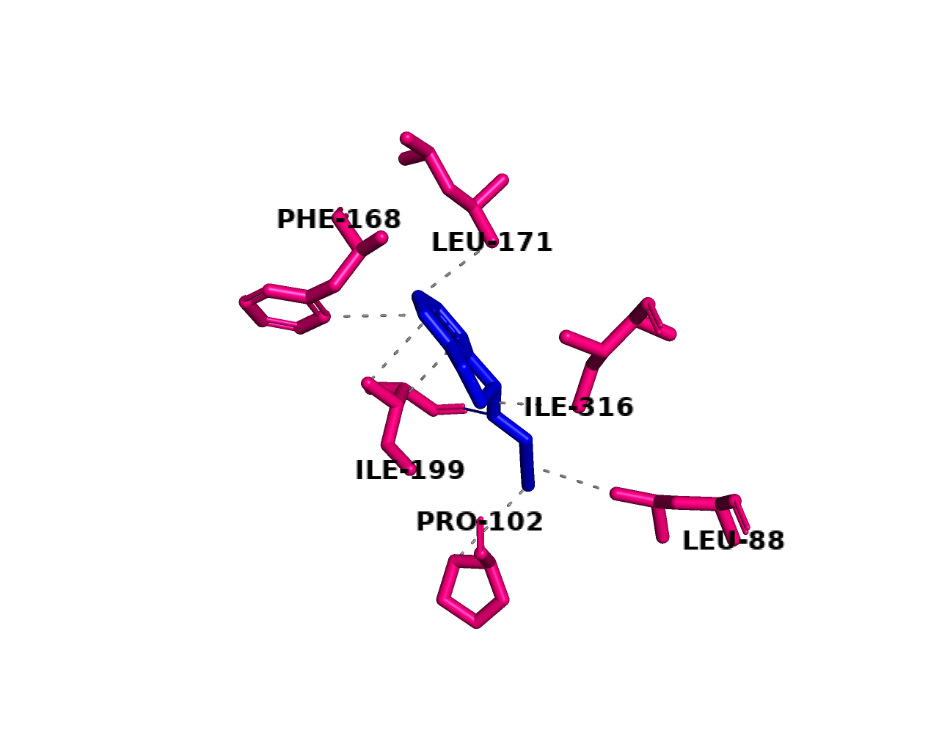


**Rasagiline-MAO-B Complex**


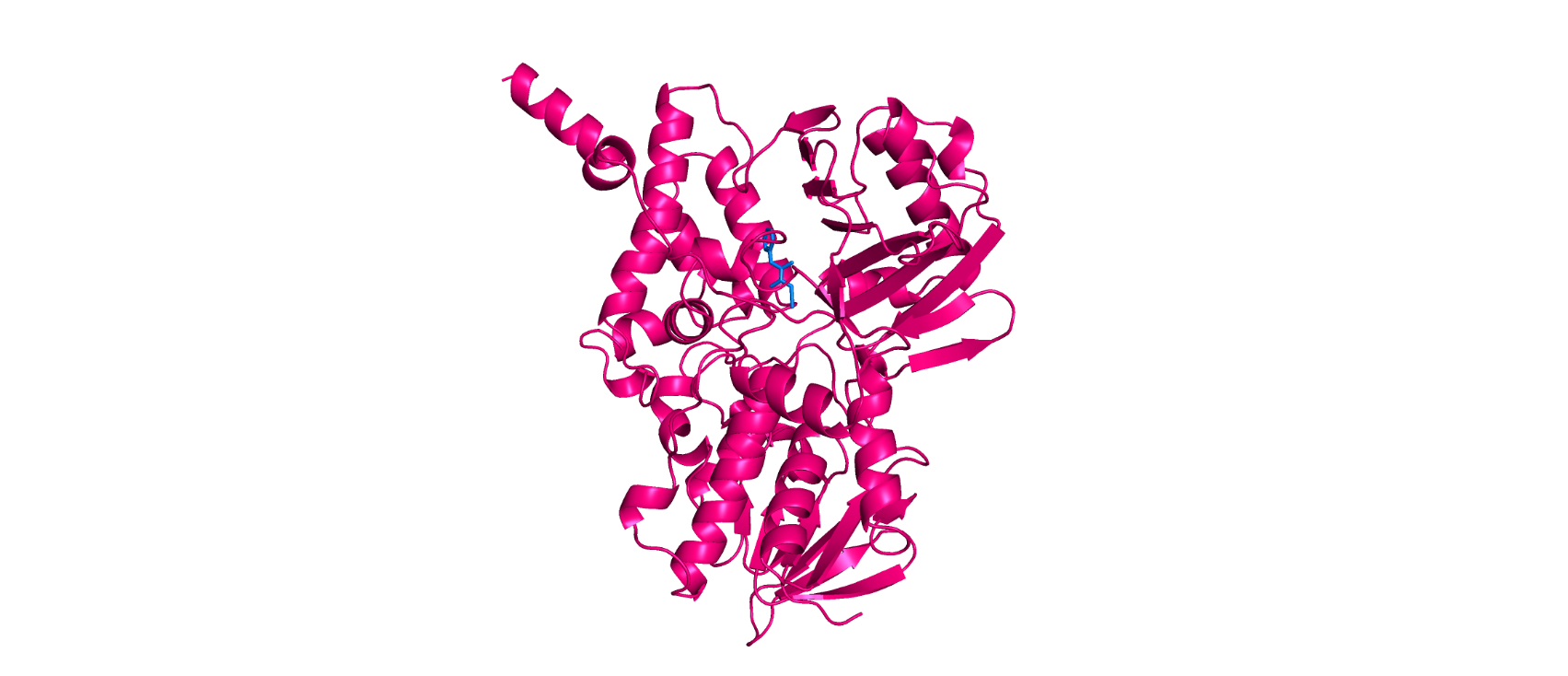

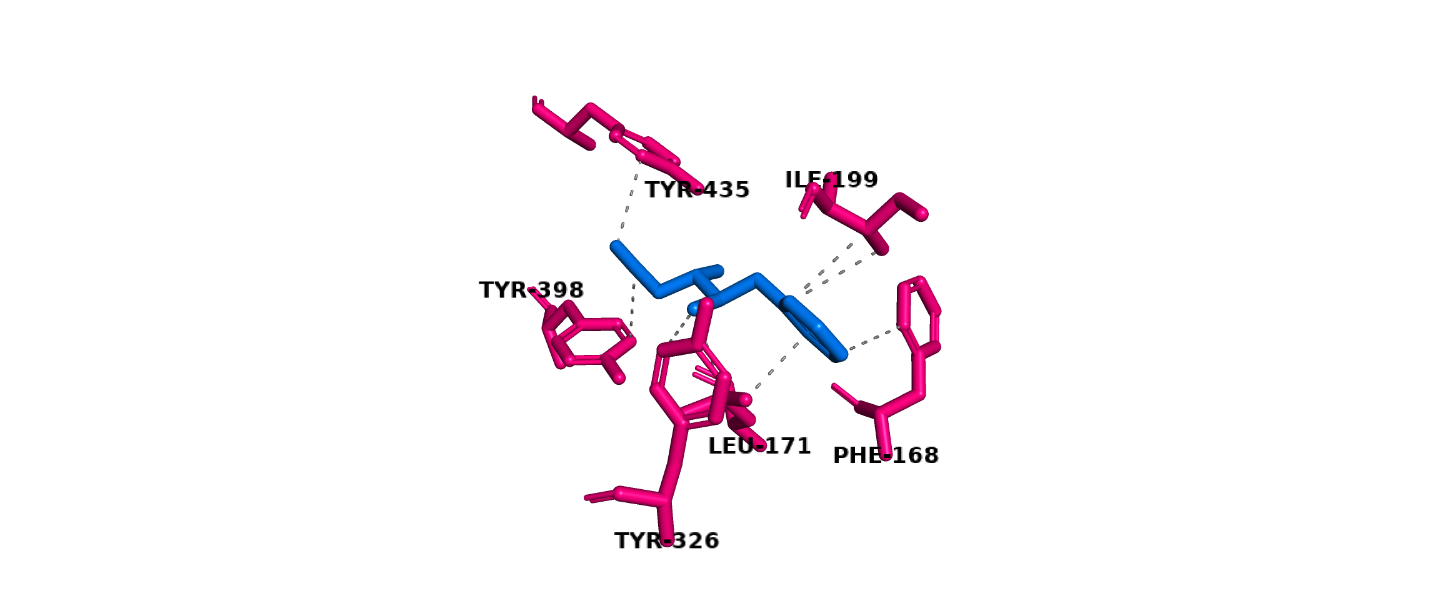


**L-deprenyl-MAO-B Complex**

**S1 Fig. Binding modes and interactions of ligands with MAO-B: Ribbon model representation (left) showing the orthosteric pocket of MAO-B (PDB ID: 4A79) and 3D interaction profiles (right) illustrating the interacting amino acid residues of the enzyme with the top 8 ligands, along with the native ligand.**


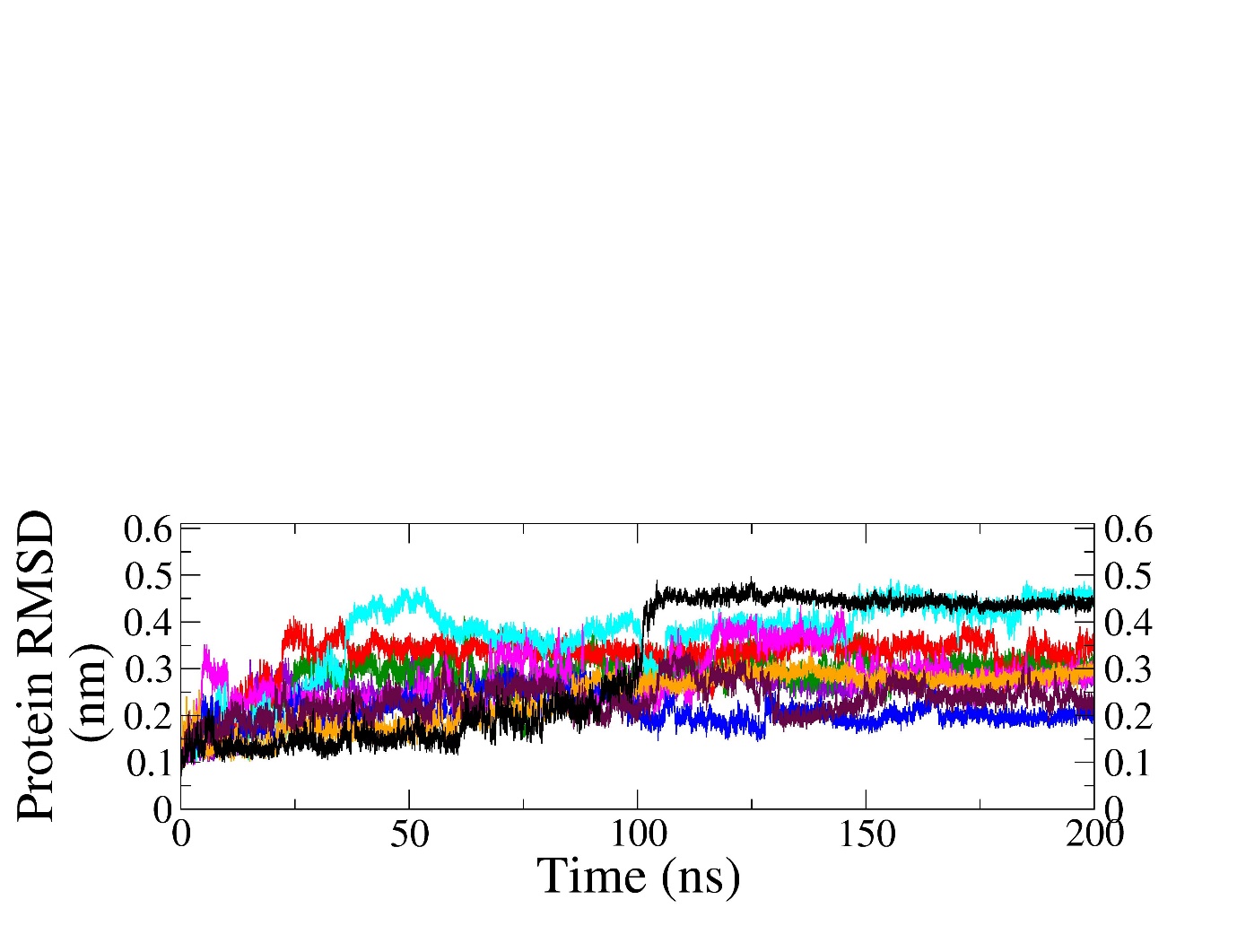


**S2 Fig. RMSD of protein backbone complexed with top 8 ligands relative to protein backbone along with apo protein (black); Complex 1; violet= beta-sitosterol, Complex 2; green= squalene, Complex 3; red= etretinate, Complex 4; cyan= rhoifolin, Complex 5; magenta= swertisin, Complex 6; blue= phloridzin, Complex 7; orange= rhapontin, Complex 8; maroon= diosmetin-7-O-beta-D-glucopyranoside.**


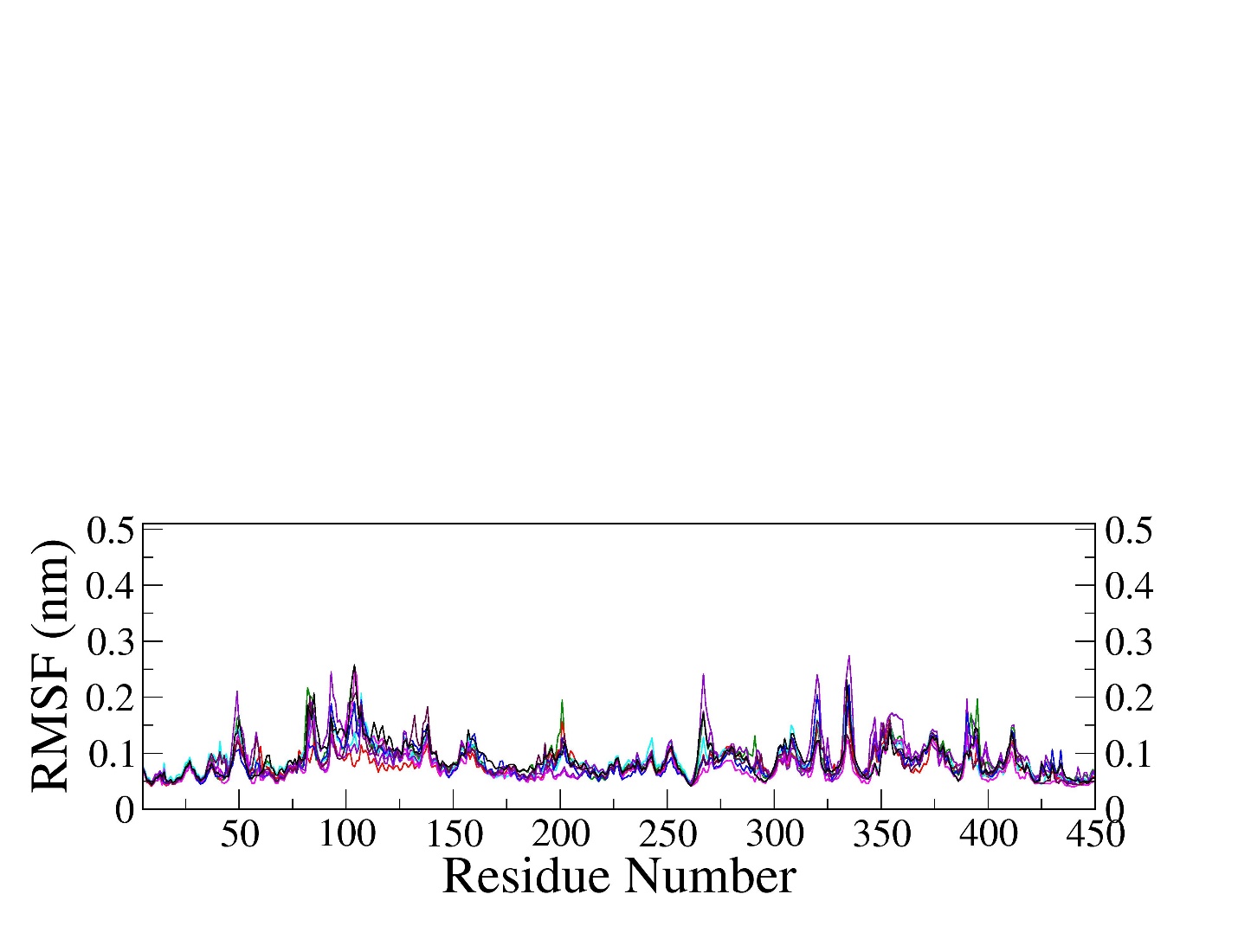


**S3 Fig. RMSF curves of alpha-carbon atoms of protein backbone in top 8 protein-ligand complexes relative to the protein backbone along with that of apo protein (black); Complex 1; violet= beta-sitosterol, Complex 2; green= squalene, Complex 3; red= etretinate, Complex 4; cyan= rhoifolin, Complex 5; magenta= swertisin, Complex 6; blue= phloridzin, Complex 7; orange= rhapontin, Complex 8; maroon= diosmetin-7-O-beta-D-glucopyranoside.**


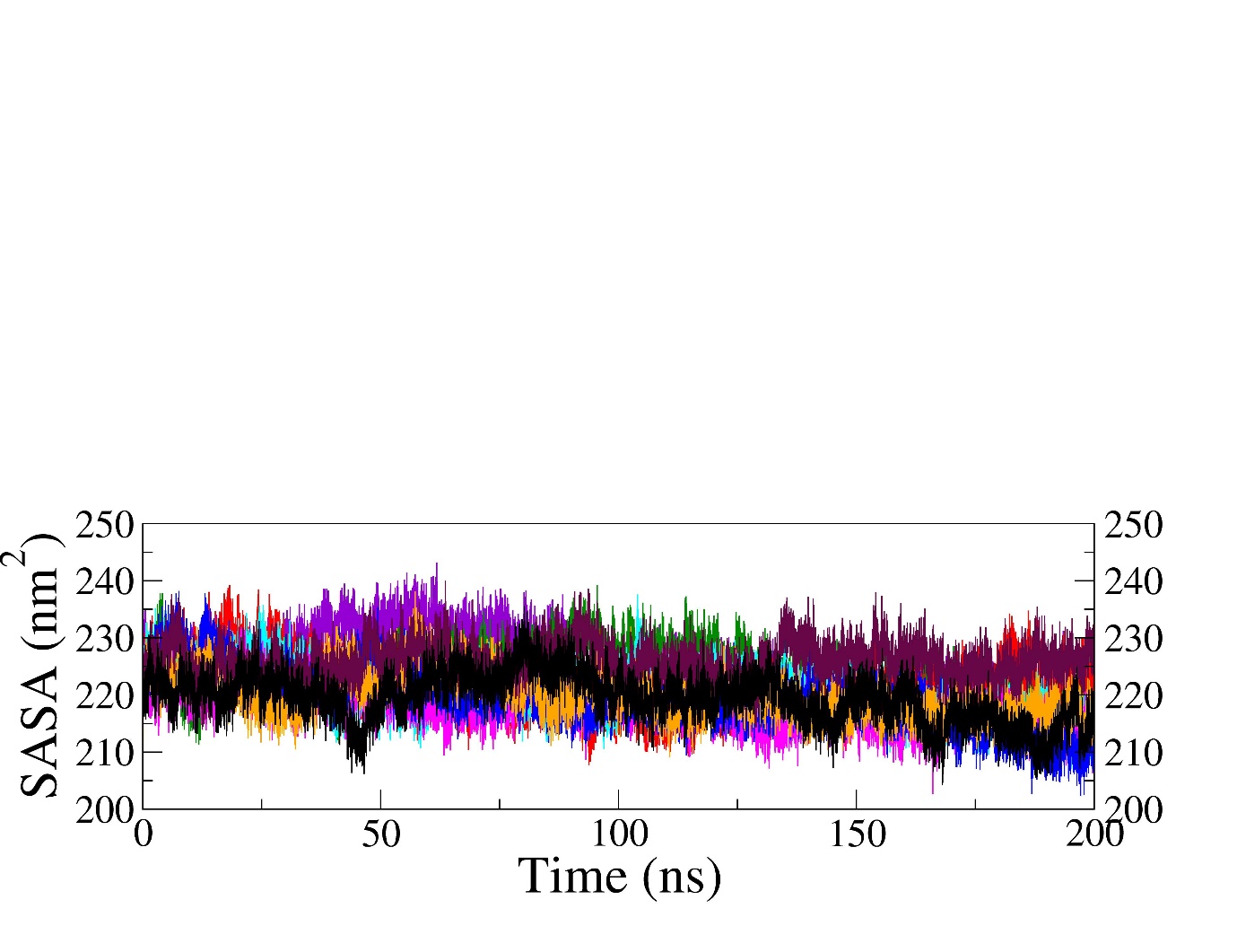


**S4 Fig. Variation of SASA of proteins in top 8 protein ligand complexes compared to that of the apo protein (black). Complex 1; violet= beta-sitosterol, Complex 2; green= squalene, Complex 3; red= etretinate, Complex 4; cyan= rhoifolin, Complex 5; magenta= swertisin, Complex 6; blue= phloridzin, Complex 7; orange= rhapontin, Complex 8; maroon= diosmetin-7-O-beta-D-glucopyranoside.**


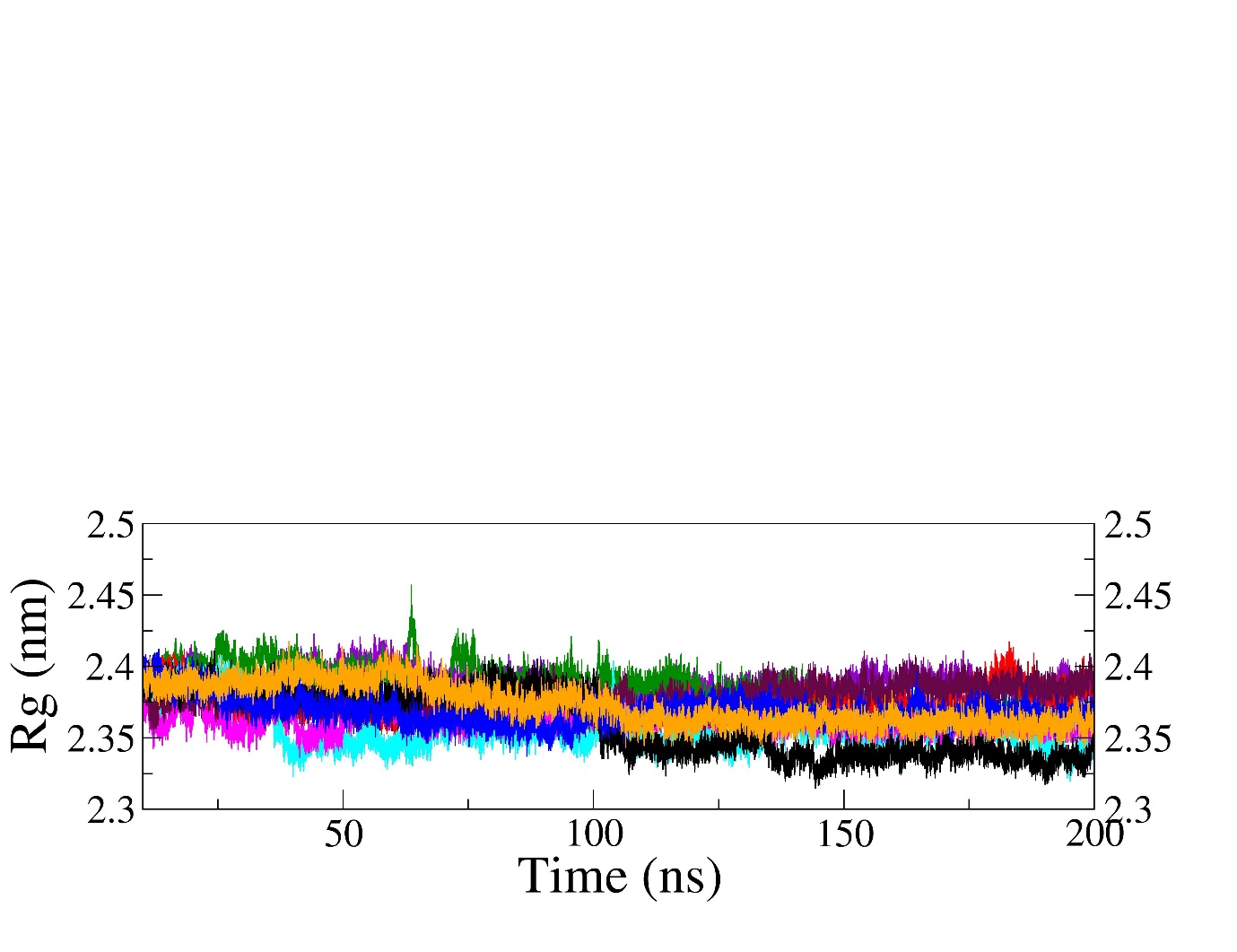


**S5 Fig. Variation of the radius of gyration of the protein in top 8 protein-ligand complexes and the apo protein (black) obtained from the MDS trajectories; Complex 1 (violet); Complex 2 (green); Complex 3 (red); Complex 4 (cyan); Complex 5 (magenta); Complex 6 (blue); Complex 7 (orange); Complex 8 (maroon).**


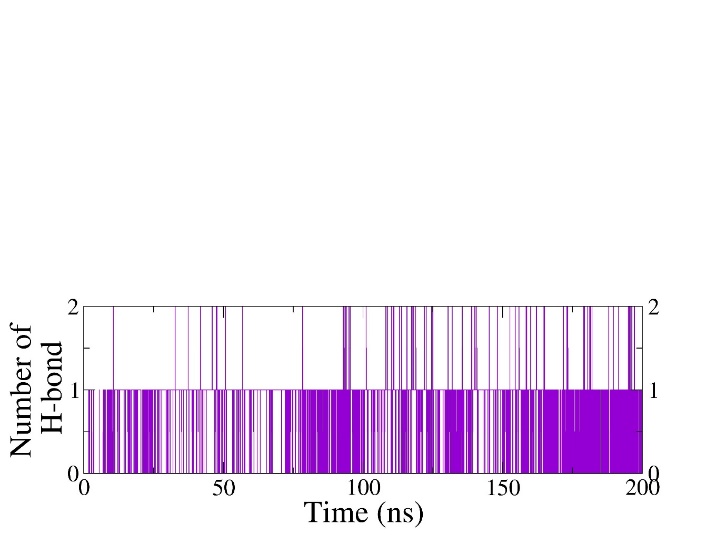

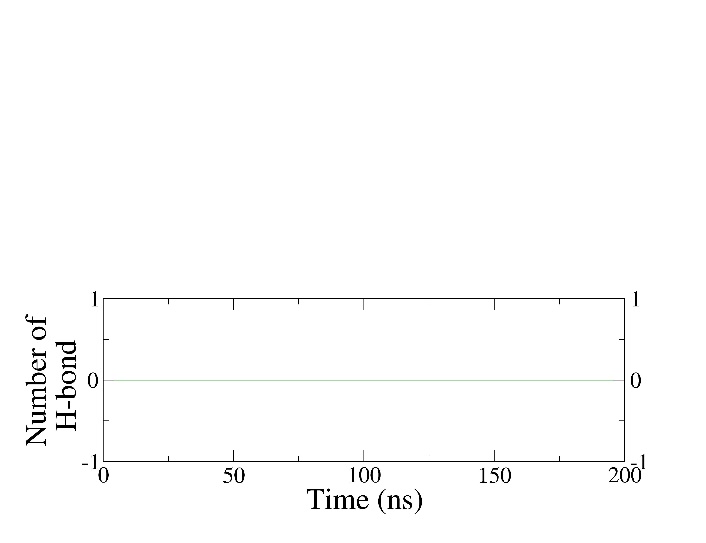


**(1) (2)**


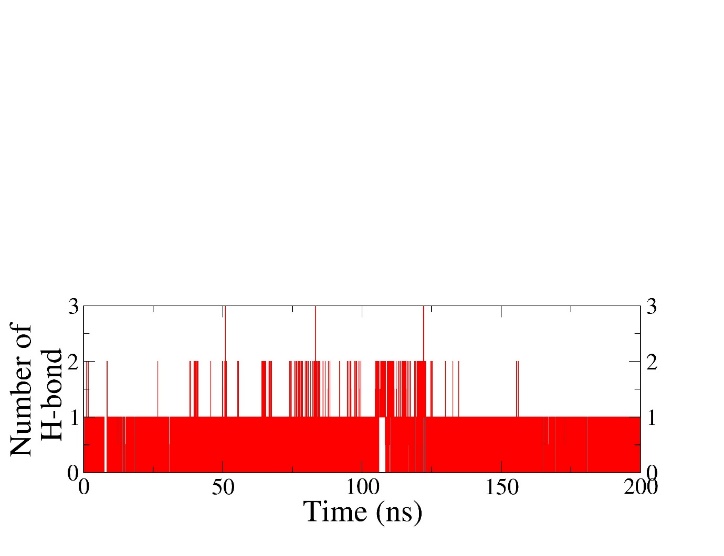

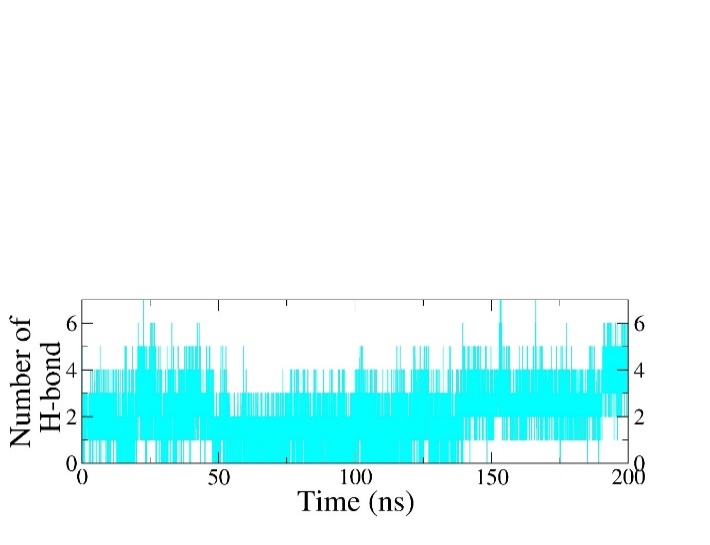


**(3) (4)**


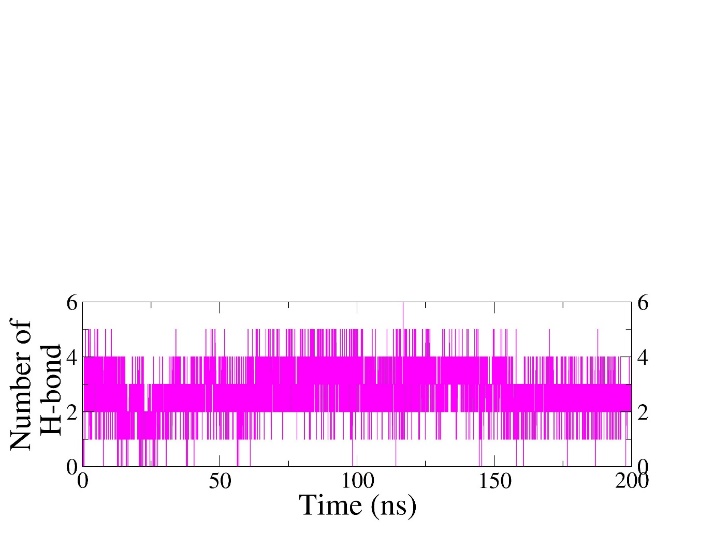

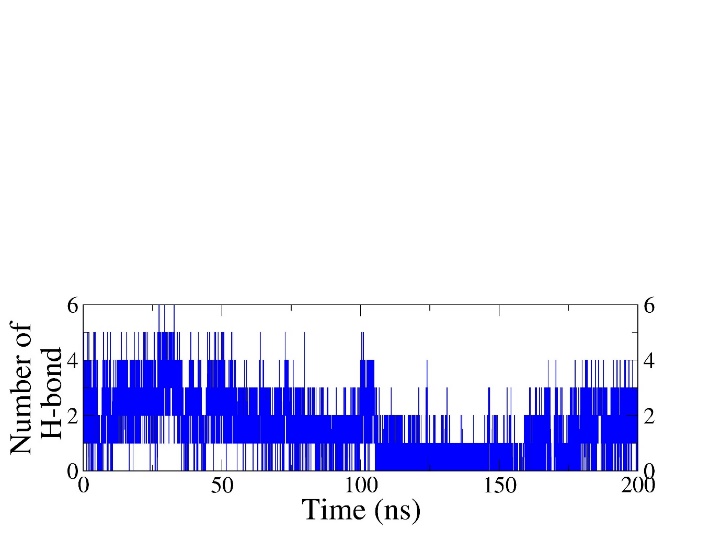


**(5) (6)**


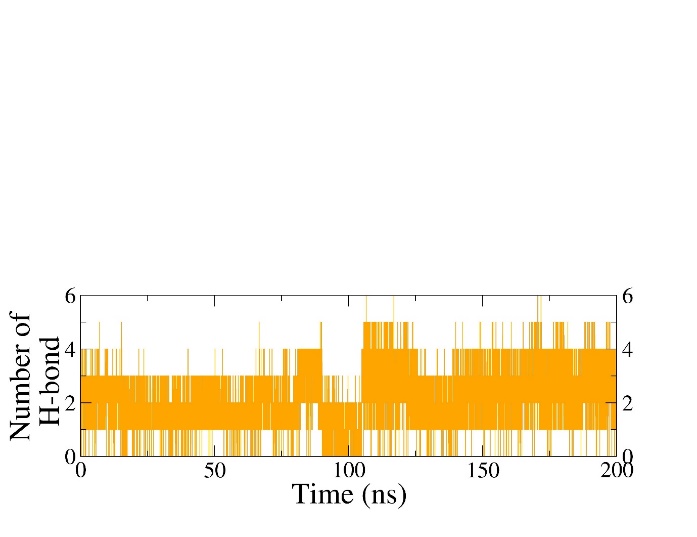

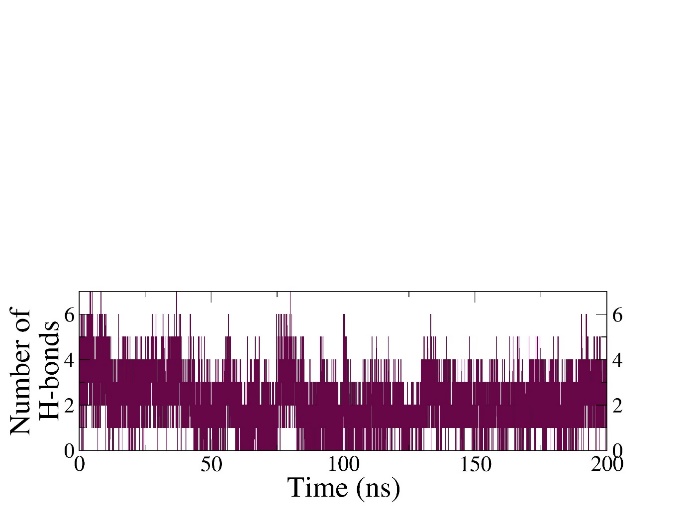


**(7) (8)**

**S6 Fig. Variation in hydrogen bond count in the top 8 protein ligand complexes throughout the MDS; (1) Complex 1 (violet); (2) Complex 2 (green)** **( H-bond absent in Complex2); (3) Complex 3 (red); (4) Complex 4 (cyan); (5) Complex 5 (magenta); (6) Complex 6 (blue); (7) Complex 7 (orange); (8) Complex 8 (maroon).**


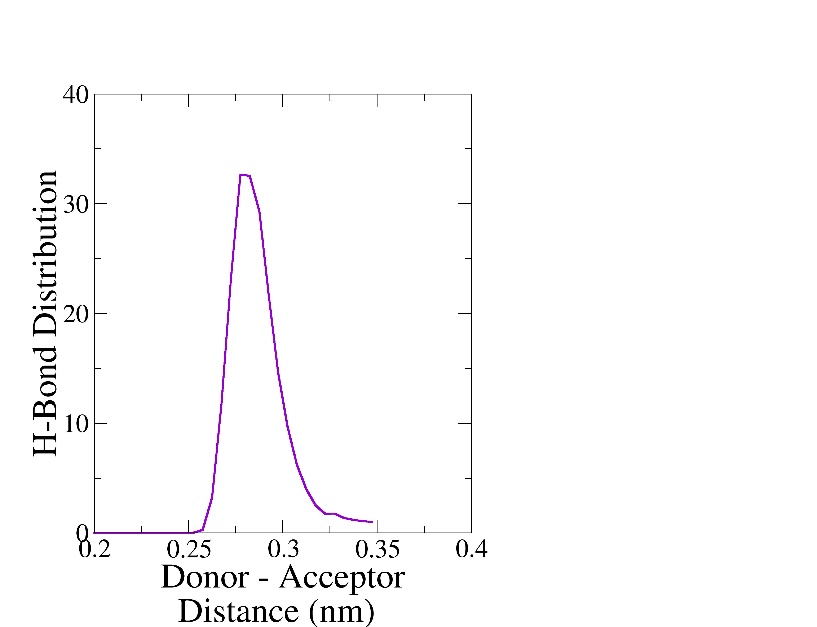

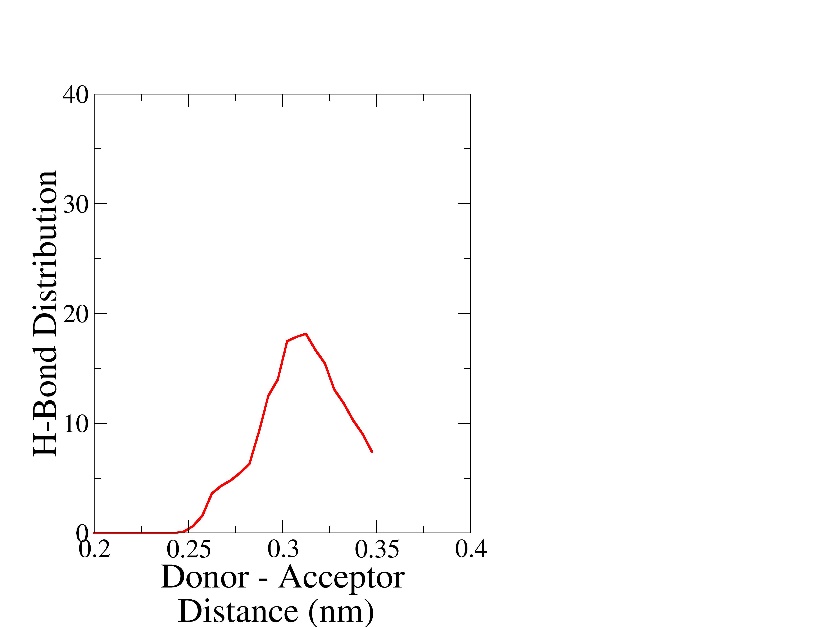


**(1)** **(3)**


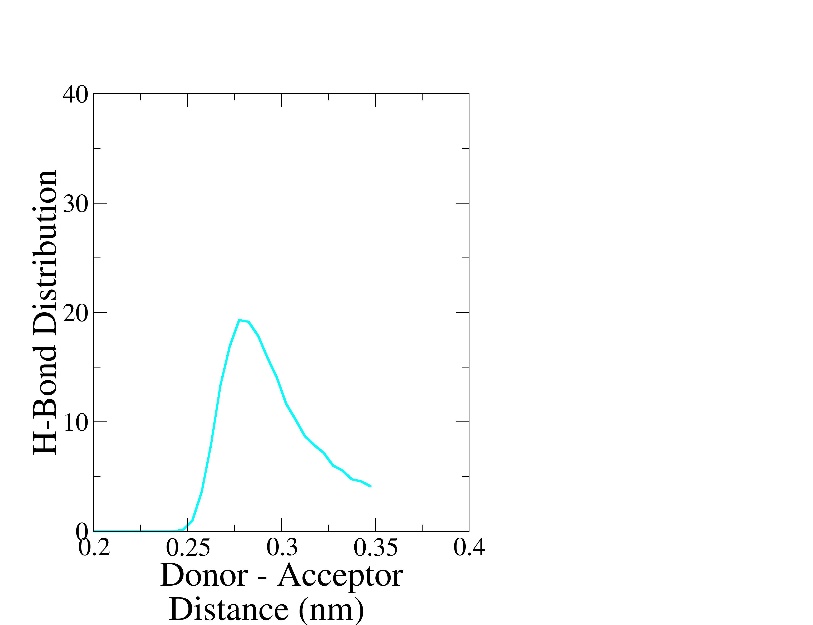

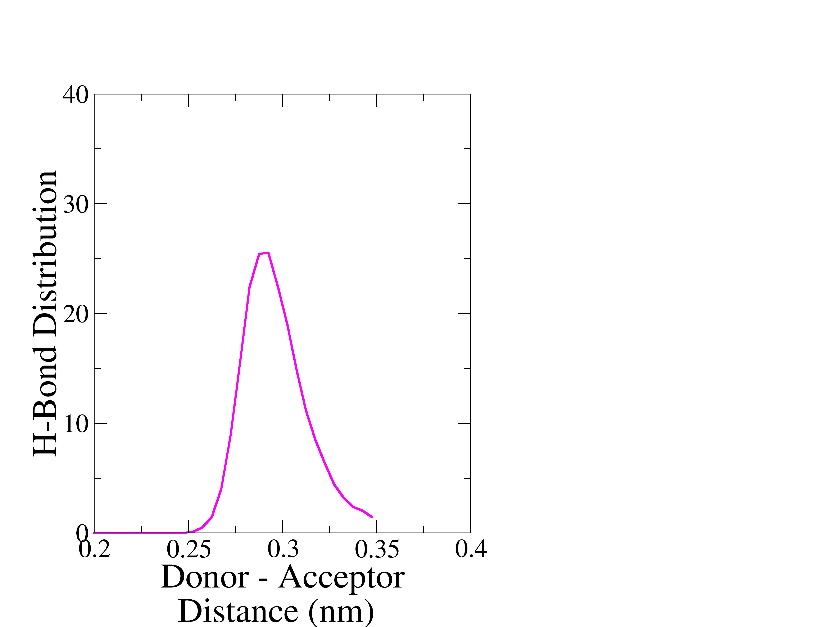


**(4)** **(5)**


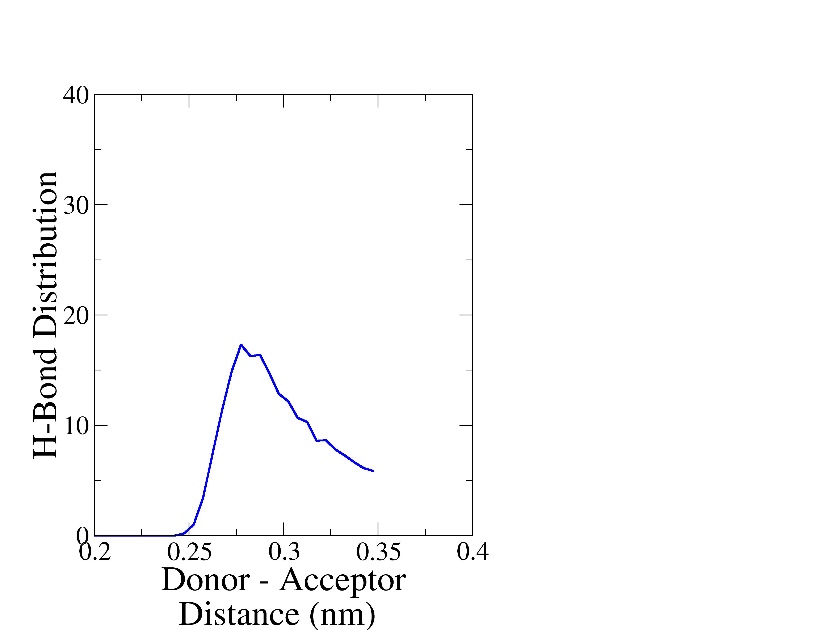

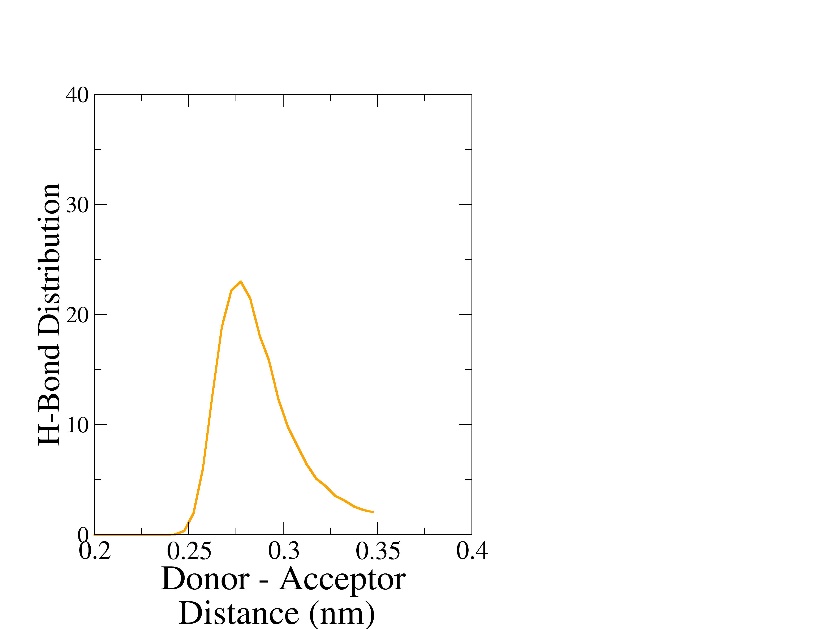


**(6)**  **(7)**


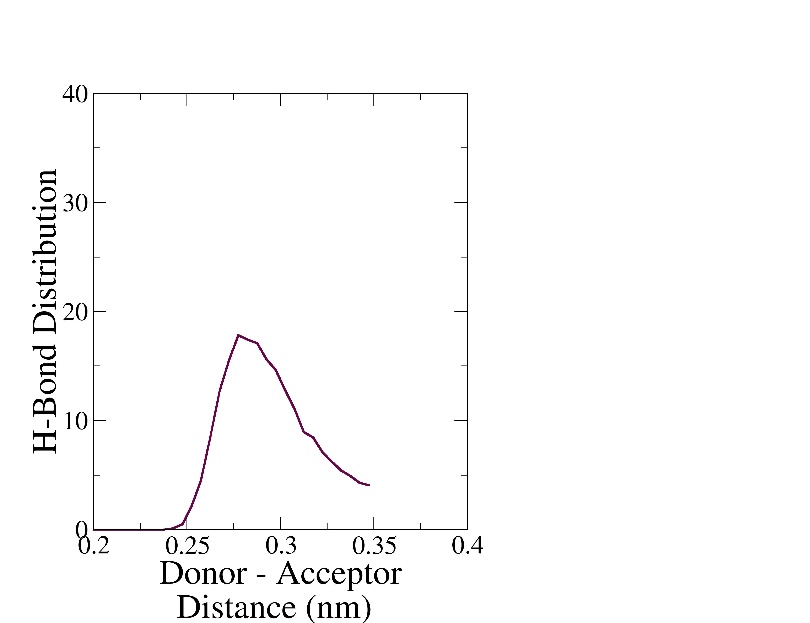


**(8)**

**S7 Fig. The frequency of donor-acceptor distance in hydrogen bonds within the top 8 protein ligand complexes (no H-Bond in Complex 2) during MDS; (1) Complex 1 (violet); (3) Complex 3 (red); (4) Complex 4 (cyan); (5) Complex 5 (magenta); (6) Complex 6 (blue); (7) Complex 7 (orange); (8) Complex 8 (maroon).**


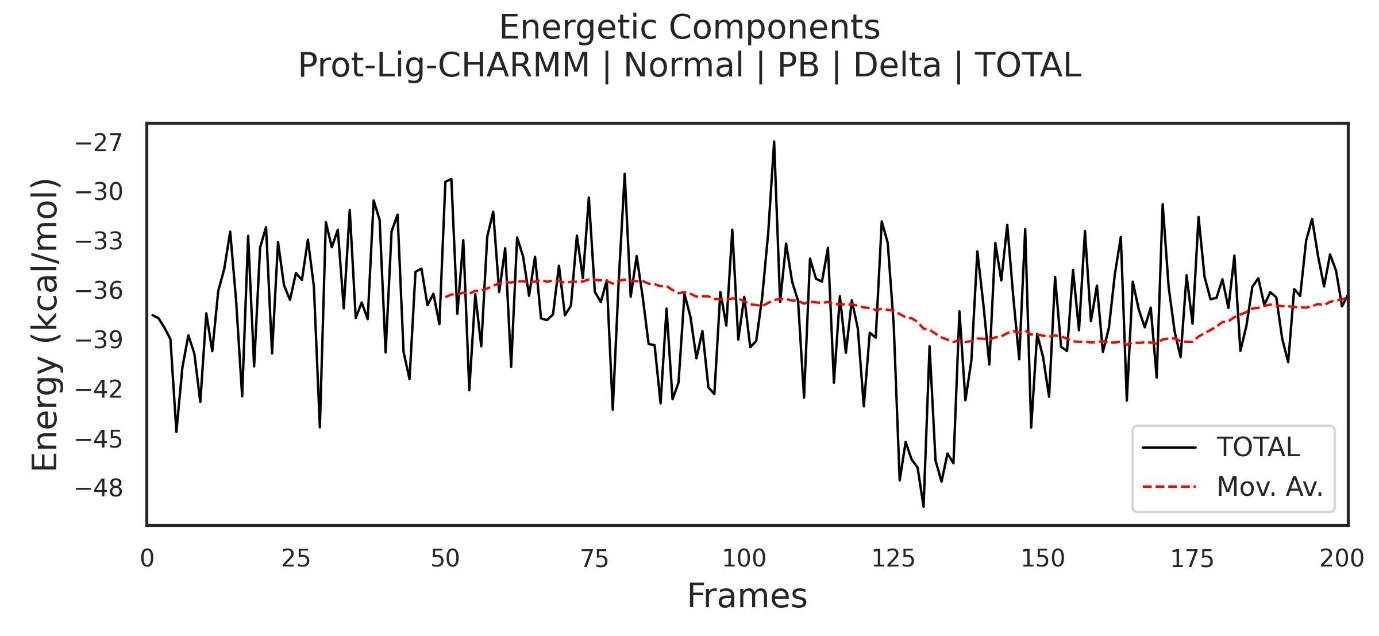


**Complex 1**


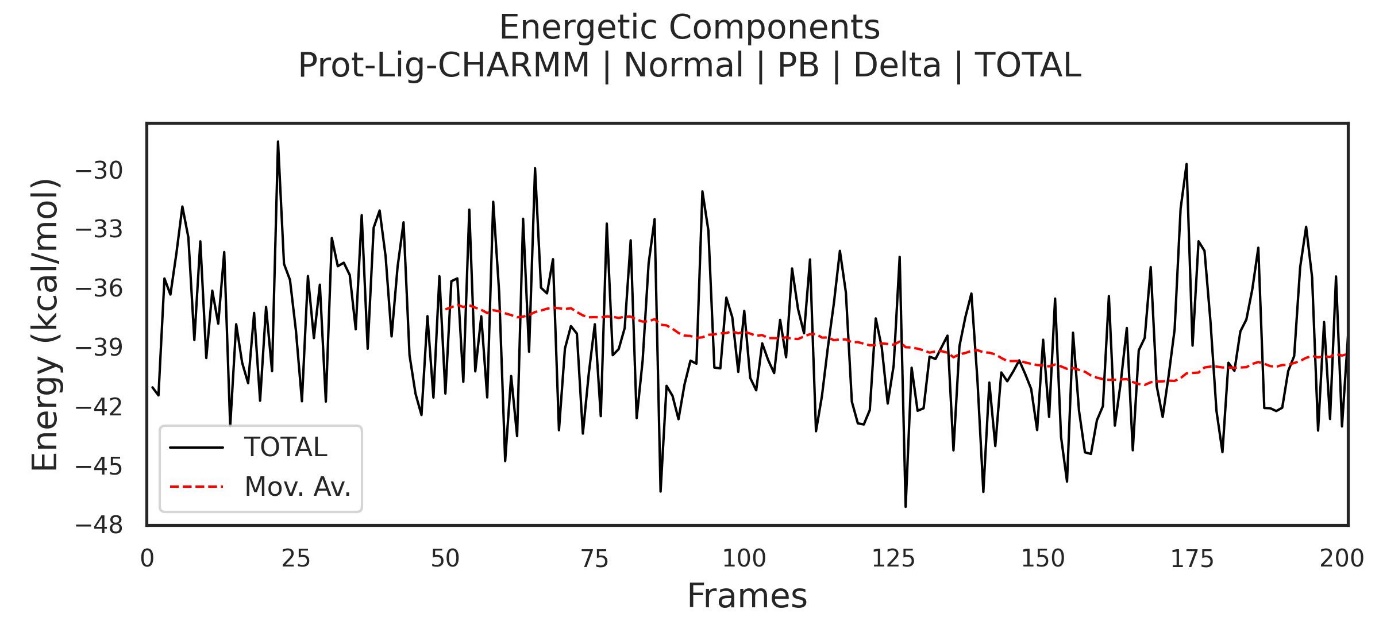


**Complex 2**


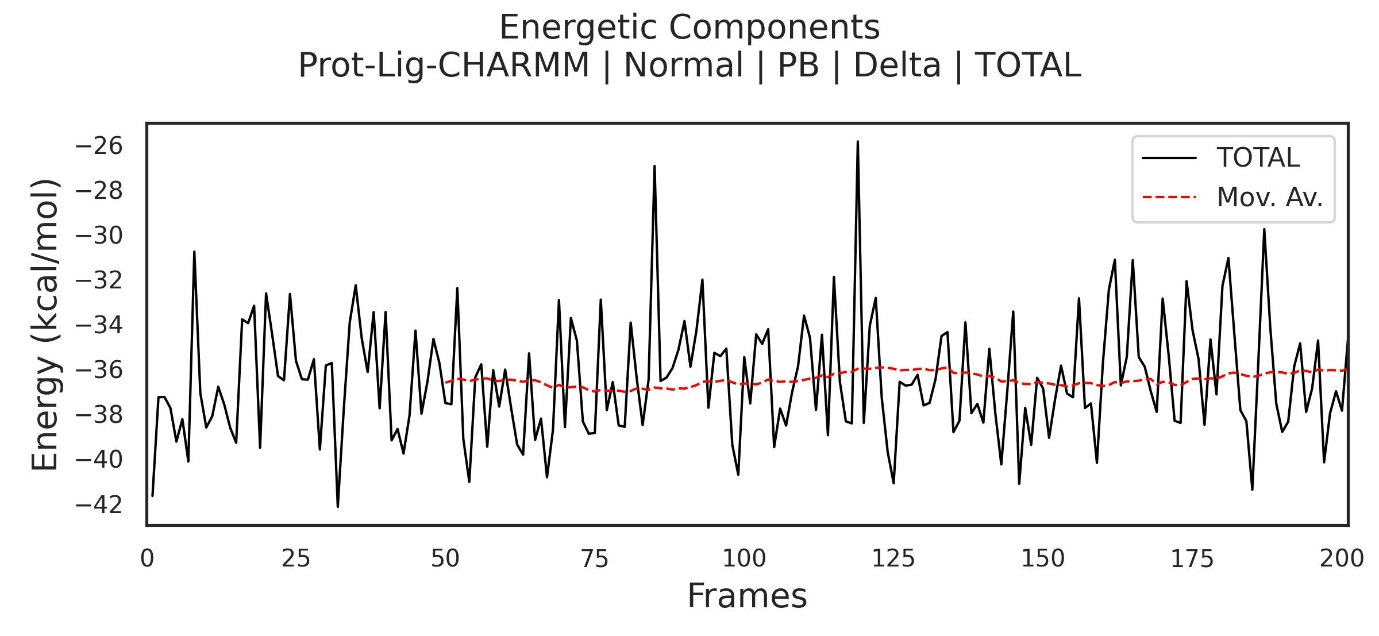


**Complex 3**


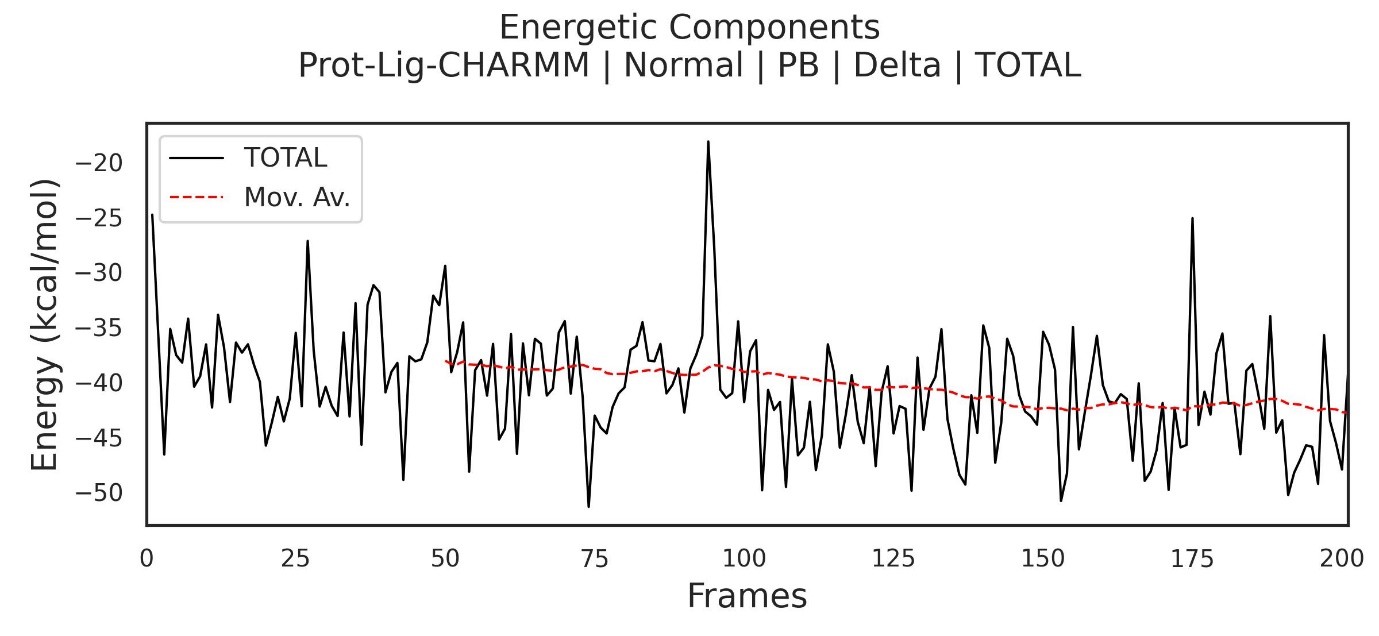


**Complex 4**


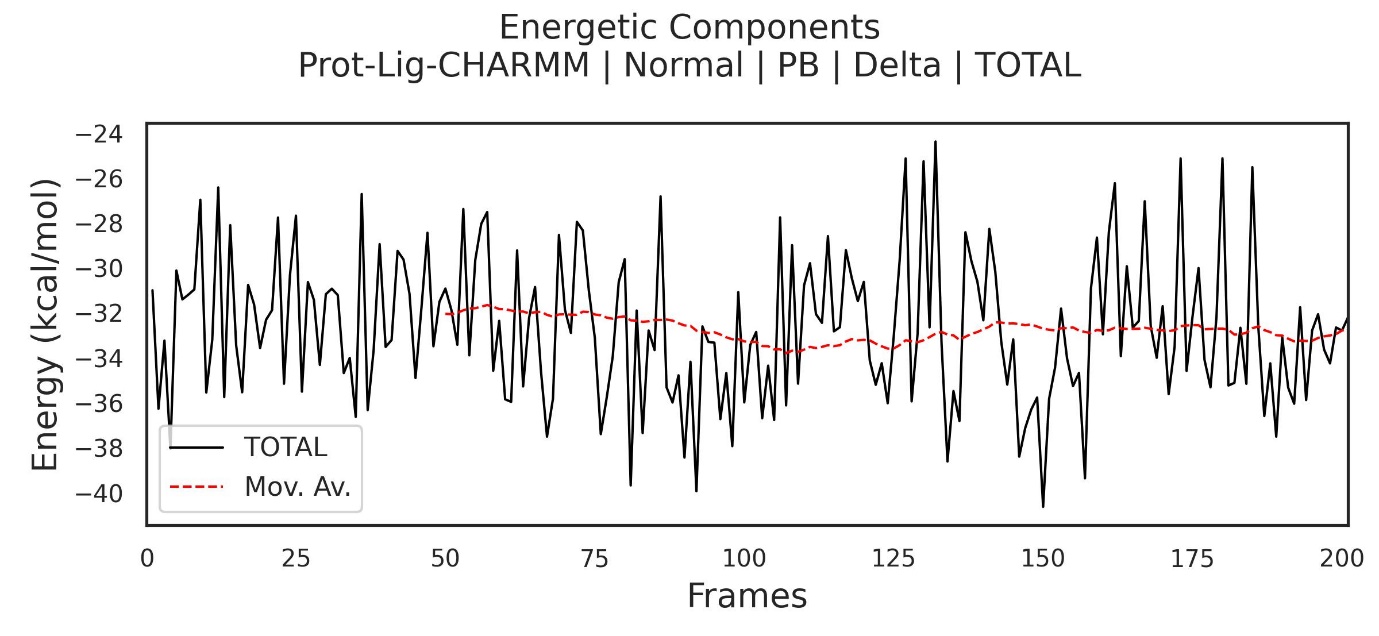


**Complex 5**


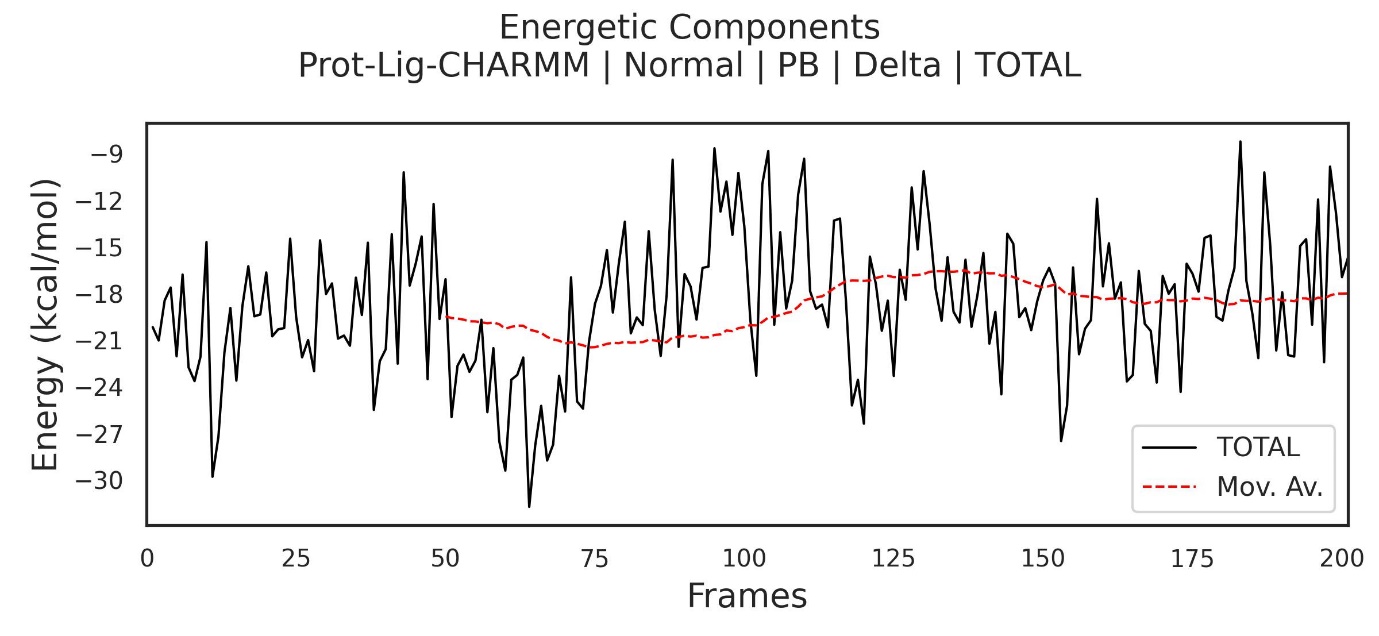


**Complex 6**


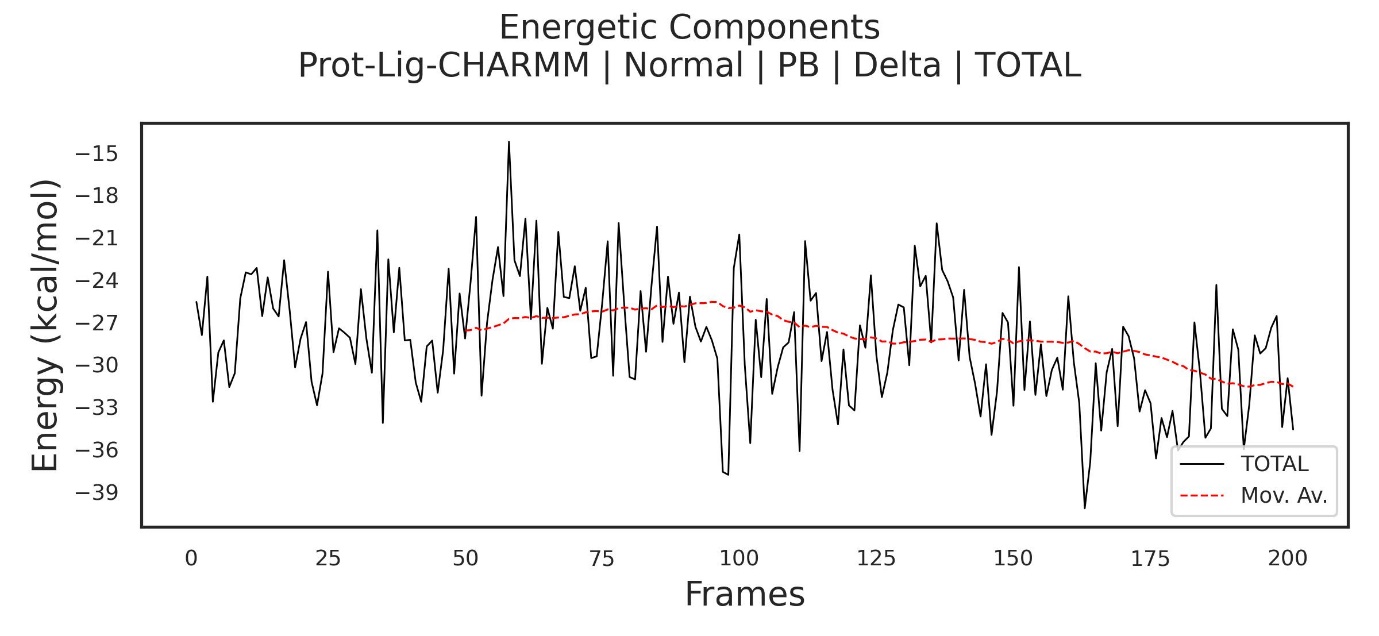


**Complex 7**
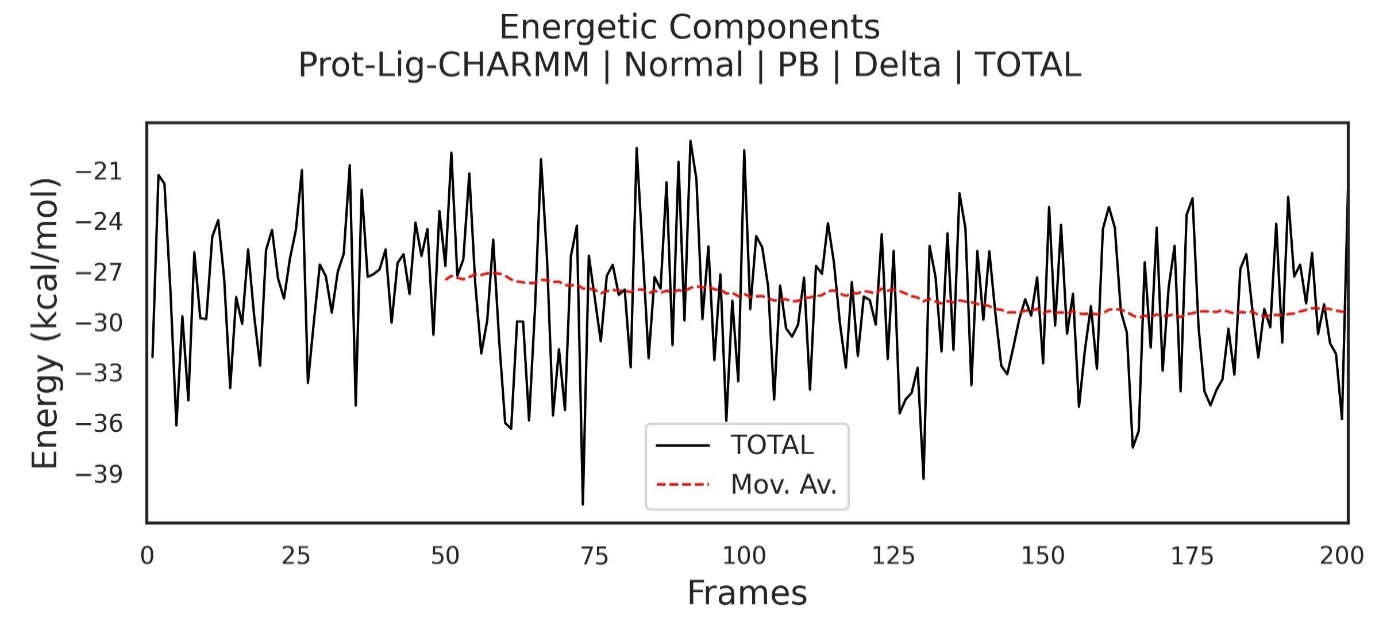


**Complex 8**

**S8 Fig. Variational curves of binding free energy change for the top 8 protein-ligand adducts across the equilibrated segment of MDS trajectories; persistently negative moving averages, confirm the steady spontaneity throughout the production run.**

**S1 Table. Binding affinity of phytocompounds present in Oxalis corniculata and Oxalis latifolia**

| **S.No.** | **Compounds** | **PubChem CID** | **Binding affinity (kcal/mol)** |
| --- | --- | --- | --- |
| 1 | Beta-Sitosterol | 222284 | −11.92 |
| 2 | Squalene | 638072 | −11.89 |
| 3 | Etretinate | 5282375 | −11.46 |
| 4 | Rhoifolin | 5282150 | −11.44 |
| 5 | Swertisin | 124034 | −11.13 |
| 6 | Phloridzin | 6072 | −11.10 |
| 7 | Rhapontin | 637213 | −11.02 |
| 8 | Diosmetin 7-O-beta-D-glucopyranoside | 11016019 | −10.96 |
| 9 | Isoorientin | 114776 | −10.95 |
| 10 | Isovitexine | 162350 | −10.91 |
| 11 | Astringin | 5281712 | −10.81 |
| 12 | Apigenin-7-O-glucoside (Cosmosiin) | 5280704 | −10.81 |
| 13 | Luteolin-7-O-glucoside | 5280637 | −10.78 |
| 14 | Cetylpyridinium | 2683 | −10.76 |
| 15 | Epirubicin | 41867 | −10.66 |
| 16 | Quercitrin | 5280459 | −10.43 |
| 17 | Methoprene (s) | 1711973 | −10.39 |
| 18 | Granisetron | 5284566 | −10.37 |
| 19 | Dihydroceramide C2 | 6610273 | −10.31 |
| 20 | Petunidin | 441774 | −10.25 |
| 21 | Orientin | 5281675 | −10.17 |
| 22 | Phytosphingosine | 122121 | −10.09 |
| 23 | Betulin | 122121 | −10.05 |
| 24 | Ophiobolin A | 5281387 | −10.03 |
| 25 | Vitexin | 5280441 | −9.94 |
| 26 | Docosanedioic Acid | 244872 | −9.91 |
| 27 | Dihydrosphingosine | 91486 | −9.84 |
| 28 | Luteolin | 5280445 | −9.69 |
| 29 | Lecanoric Acid | 99613 | −9.66 |
| 30 | Apigenin | 5280443 | −9.66 |
| 31 | Naringenin | 439246 | −9.50 |
| 32 | Embelin | 3218 | −9.49 |
| 33 | Dibekacin | 470999 | −9.15 |
| 34 | Parthenin | 442288 | −9.04 |
| 35 | Methyclothiazide | 4121 | −8.39 |
| 36 | Sebacic Acid | 5192 | −7.70 |

**S2 Table. Physiochemical properties of the top ligands along with that of native ligand and reference drugs**

| **Drug-likeness**  **parameters**  **Compounds** | | **Mol. Wt. (g/mol)** | **NRB** | **NHA** | **NHD** | **LogP** | **Lipinski’s Rule (RO5)** |
| --- | --- | --- | --- | --- | --- | --- | --- |
| **Top ligands** | Beta-sitosterol | 414.71 | 6 | 1 | 1 | 8.02 | Accepted |
|  | Squalene | 410.73 | 15 | 0 | 0 | 10.60 | Accepted |
|  | Etretinate | 354.49 | 7 | 3 | 0 | 5.64 | Accepted |
|  | Rhoifolin | 578.52 | 6 | 14 | 8 | -1.09 | Rejected |
|  | Swertisin | 446.40 | 4 | 10 | 10 | 0.39 | Accepted |
| **Native** | P1B, Pioglitazone | 356.44 | 7 | 5 | 1 | 3.15 | Accepted |
| **Reference drugs** | Safinamide | 302.34 | 7 | 3 | 2 | 2.36 | Accepted |
|  | L-Deprenyl | 187.28 | 4 | 1 | 0 | 2.18 | Accepted |
|  | Rasagiline | 171.24 | 2 | 1 | 1 | 1.89 | Accepted |

Note: NRB- No. of Rotatable Bonds; NHA- No. of Hydrogen Acceptor; NHD- No. of Hydrogen Donors

**S3 Table. ADMET profiling of top five ligands along with that of native ligand and reference drugs considered.**

| **ADMET parameters** | | **Hit ligands** | | | | | **Native ligand** | **Reference drugs** | | |
| --- | --- | --- | --- | --- | --- | --- | --- | --- | --- | --- |
|  |  | **Beta-sitosterol** | **Squalene** | **Etretinate** | **Rhoifolin** | **Swertisin** | **P1B** | **Safinamide** | **L-deprenyl** | **Rasagiline** |
| **A** | **HIA absorption (%)** | 94.46 | 89.00 | 95.94 | 24.30 | 50.84 | 96.762 | 94.01 | 92.57 | 98.65 |
|  | **P-gp substrate** | No | No | No | Yes | Yes | Yes | Yes | No | Yes |
| **D** | **BBB (log BB)** | 0.78 | 0.96 | 0.23 | −1.70 | −1.56 | −0.49 | −0.38 | 1.01 | 0.73 |
|  | **CNS permeability (log PS)** | −1.70 | −0.93 | −1.84 | −4.79 | −3.90 | −2.48 | −2.70 | −1.98 | −1.74 |
| **M** | **CYP1A2 inhibitor** | No | No | No | No | No | Yes | No | Yes | Yes |
|  | **CYP2C19 inhibitor** | No | No | No | No | No | Yes | No | No | No |
|  | **CYP2C9 inhibitor** | No | No | No | No | No | No | No | No | No |
|  | **CYP2D6 inhibitor** | No | No | No | No | No | No | No | No | No |
|  | **CYP3A4 inhibitor** | No | No | No | No | No | No | No | No | No |
| **E** | **Total Clearance (log ml/min/kg)** | 0.62 | 1.79 | 0.96 | −0.01 | 0.48 | −0.04 | 0.97 | 1.01 | 1.22 |
| **T** | **AMES toxicity** | No | No | No | No | No | No | No | No | No |
|  | **Max. tolerated dose (human) (log mg/kg/day)** | −0.62 | -0.53 | 0.66 | 0.49 | 0.64 | 0.85 | 0.61 | 0.19 | 1.16 |
|  | **hERG I inhibitor** | No | No | No | No | No | No | No | No | No |
|  | **Oral rat acute toxicity (LD_50_ = mol/kg)** | 2.55 | 1.89 | 2.13 | 2.49 | 2.55 | 2.487 | 1.98 | 3.09 | 2.23 |
|  | **Hepatotoxicity** | No | No | Yes | No | No | Yes | Yes | Yes | No |
|  | **Skin sensitization** | No | No | No | No | No | No | No | Yes | Yes |

Note: HIA: - Human Intestinal Absorption, P-gp: - P-glycoprotein, BBB: - Blood Brain Barrier, CNS: - Central Nervous System, CYP: - Cytochrome P450, hERG: - human Ether-a-go-go-Related Gene

**Molecular Docking Input Files**

INPUT FILES

protein = protein_1375de5.in

ligand set size = 36

ligand files: ligand_5cd255; ligand_ff37577a; ligand_73ff08b847; ligand_67cec4d068; ligand_f1c3118ea7; ligand_0f4d3996db; ligand_844b0e; ligand_431181c7d; ligand_97ee939; ligand_0a695e27; ligand_cec9f7ac46; ligand_8da1073dec; ligand_720fd47a0c; ligand_f757ed0351; ligand_d3bbf45121; ligand_f7341de4; ligand_27f7090786; ligand_8e348ef642; ligand_af6f61d907; ligand_109cd5; ligand_be6f3f9e; ligand_a3e7f71f25; ligand_9d941ee8c3; ligand_175d06c77a; ligand_2ae8686f7e; ligand_a44b2a640; ligand_1c10b7833c; ligand_e36327d275; ligand_65792961d7; ligand_07a28f4116; ligand_47113cc7de; ligand_c0d7f439e4; ligand_3aead1878a; ligand_a576e68c36; ligand_8c0cf9c; ligand_b65bad3952

cofactor set size = 0

cofactor files =

GRID SETTINGS

center x = 51

center y = 157

center z = 31

total size x = 15

total size y = 15

total size z = 15

discretization = 0.16

GENETIC ALGORITHM SETTINGS

number of evaluations = 1000000

population size = 750

number of runs = 24

seed at run #1 = -1985

**Molecular Dynamics Simulation Input Files**

**1. md.mdp**

title = Protein-ligand complex MDS

; Run parameters

integrator = md ; leap-frog integrator

nsteps = 100000000 ; 2 * 100000000 = 200000 ps (200 nanoseconds)

dt = 0.002 ; 2 femtosecond

; Output control

nstenergy = 5000 ; save energies every ten ps

nstlog = 5000 ; update log file every ten ps

nstxout-compressed = 5000 ; save coordinates every ten ps

; Bond parameters

continuation = yes ; continuing from NPT equilibration

constraint_algorithm = lincs ; holonomic constraints

constraints = h-bonds ; bonds to H are constrained

lincs_iter = 1 ; accuracy of LINCS

lincs_order = 4 ; also related to accuracy

; Neighbor searching and vdW

cutoff-scheme = Verlet

ns_type = grid ; search neighboring grid cells

nstlist = 320 ; largely irrelevant with Verlet

rlist = 1.2

vdwtype = cutoff

vdw-modifier = force-switch

rvdw-switch = 1.0

rvdw = 1.2 ; short-range van der Waals cutoff (in nm)

; Electrostatics

coulombtype = PME ; Particle Mesh Ewald for long-range electrostatics

rcoulomb = 1.2

pme_order = 4 ; cubic interpolation

fourierspacing = 0.16 ; grid spacing for FFT

; Temperature coupling

tcoupl = V-rescale ; modified Berendsen thermostat

tc-grps = Protein_LIG Water_and_ions ; two coupling groups - more accurate

tau_t = 0.1 0.1 ; time constant, in ps

ref_t = 310 310 ; reference temperature, one for each group, in K

; Pressure coupling

pcoupl = Parrinello-Rahman ; pressure coupling is on for NPT

pcoupltype = isotropic ; uniform scaling of box vectors

tau_p = 2.0 ; time constant, in ps

ref_p = 1.0 ; reference pressure, in bar

compressibility = 4.5e-5 ; isothermal compressibility of water, bar^-1

; Periodic boundary conditions

pbc = xyz ; 3-D PBC

; Dispersion correction is not used for proteins with the C36 additive FF

DispCorr = no

; Velocity generation

gen_vel = no ; continuing from NPT equilibration
