# Supplementary material for: Evaluating the inhibitory efficacy of Oxalis phytocompounds on monoamine oxidase B: An integrated approach targeting age related neurodegenerative diseases through molecular docking and dynamics simulations
Source: PLoS One. 2025 Jul 30;20(7):e0329168. doi: 10.1371/journal.pone.0329168 (PMC12309996; doi:10.1371/journal.pone.0329168)
Supplement: Attachment — Submitted filename: Response_to_Reviewers.pdf [file pone.0329168.s005.pdf]

Dear Editor,

We would like to thank you and the reviewers again for the constructive and valuable feedback. We have attempted to incorporate all the suggestions to the best of knowledge and skills. It has helped to fix the errors and fulfill the insufficiency to uplift the scientific content of the manuscript. Separate images in TIF format have been included in this response letter also.

Please let us know in case the responses are unclear or insufficient. The point-by-point responses are mentioned below.

Sincerely,

Authors

Evaluating the inhibitory efficacy of *Oxalis* phytocompounds on monoamine oxidase B: An integrated approach targeting age related neurodegenerative diseases through molecular docking and dynamics simulations

JOURNAL REQUIREMENTS:

Please review your reference list to ensure that it is complete and correct. If you have cited papers that have been retracted, please include the rationale for doing so in the manuscript text, or remove these references and replace them with relevant current references. Any changes to the reference list should be mentioned in the rebuttal letter that accompanies your revised manuscript. If you need to cite a retracted article, indicate the article's retracted status in the References list and also include a citation and full reference for the retraction notice.

Response: We have thoroughly reviewed the reference list to ensure its accuracy and completeness. All cited references have been verified for current validity, and no retracted articles have been included in the manuscript. Accordingly, no changes related to retracted literature were necessary. If any further clarification is needed, we would be happy to provide additional information.

ADDITIONAL EDITOR COMMENTS:

Kudos to the authors for responding positively to the initial queries. No doubt, the quality of the submission has improved significantly. However, some concerns have been raised affecting some sections of the manuscript. I hereby recommend another round of revision to address the current concerns and reserve my final decision until they are resolved.

Response: We sincerely thank the Editor for the encouraging feedback and for acknowledging the improvements made in the revised manuscript. We appreciate the opportunity for another round of revision and have carefully addressed all the remaining concerns raised by the reviewers. The manuscript has been updated accordingly, and a detailed point-by-point response to each comment has been provided. We hope that the current version meets the expectations.

Reviewer 1:

In this research, objective was to tackle the increasing incidence of neurodegenerative disorders such as Alzheimer's and Parkinson's, which are becoming more prevalent in older populations. Acknowledging the essential function of monoamine oxidase B (MAO-B) in the metabolism of dopamine and the management of oxidative stress, authors examined plant-based compounds from *Oxalis* species as safer and more affordable alternatives to traditional MAO-B inhibitors. While current synthetic medications may be effective, they frequently come with safety issues

and high costs. Utilizing advanced computational methods, we assessed the therapeutic potential of these natural compounds. Through this research, authors highlight the importance of combining traditional medicinal practices with contemporary drug discovery technologies to facilitate the development of natural, neuroprotective approaches for neurological disorders associated with aging.

Please address following concerns:

1. I am unable to access the Figure 1. Please provide figure 1 as mentioned in Line 93. Similarly, I can’t see the figure 2, 3, 4 and 5.

Response: We apologize for the inconvenience caused by the missing figures. All figures (Fig 1–5) were included in the revised submission as per the PLOS ONE formatting guidelines. It is possible that the figures generated using the PACE server, as recommended by PLOS ONE, may have appeared unreadable or failed to render properly due to a technical issue during the file upload process or in the reviewer’s display. To address this, we have thoroughly reviewed the submission system and re-uploaded all figures, ensuring they are correctly labeled, properly formatted, and fully accessible. For clarity and to facilitate the review process, we have also

included all five figures in TIF format directly below in this response letter.

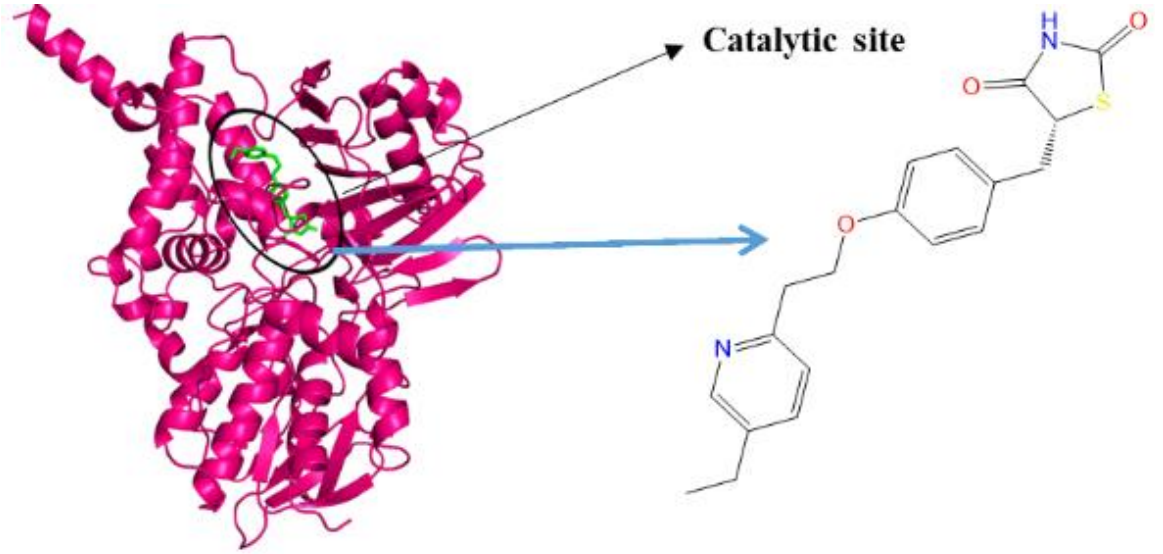

Fig 1. Monoamine Oxidase-B (MAO-B) enzyme structure in its holo form depicted in ribbon representation, with native ligand P1B, Pioglitazone, shown in bond line model at the orthosteric site.

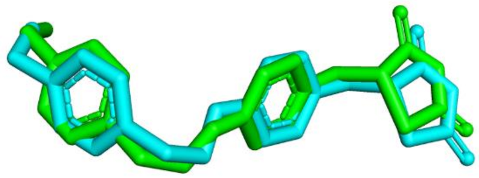

Fig 2. Superimposition of docked ligand (green) derived from the molecular docking calculations, with the native ligand (cyan) present in the crystal structure (heavy atom RMSD =0.768 Å).

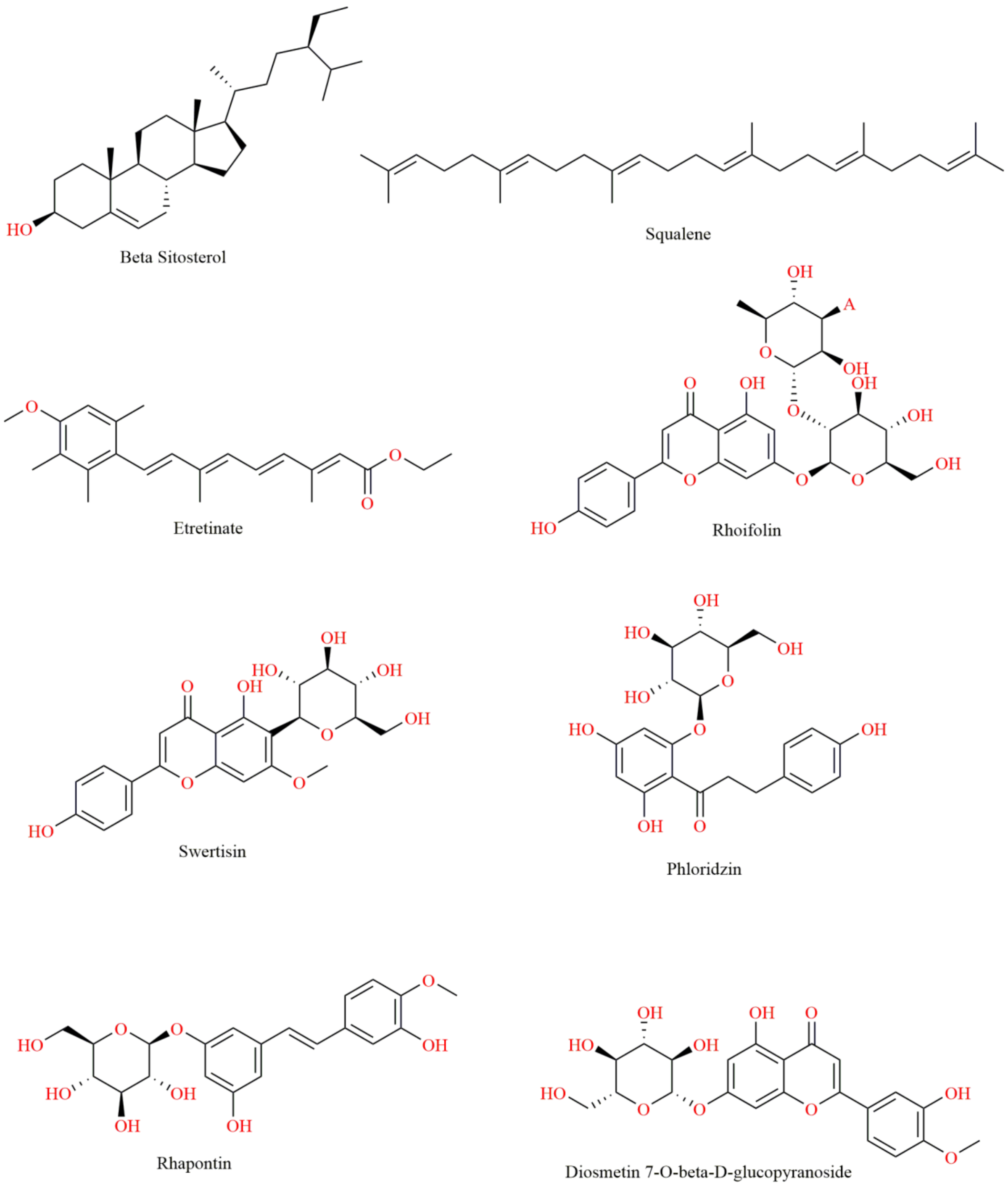

Fig 3. Chemical structures of top 8 phytochemicals from Oxalis species identified based on molecular docking scores against MAO-B enzyme

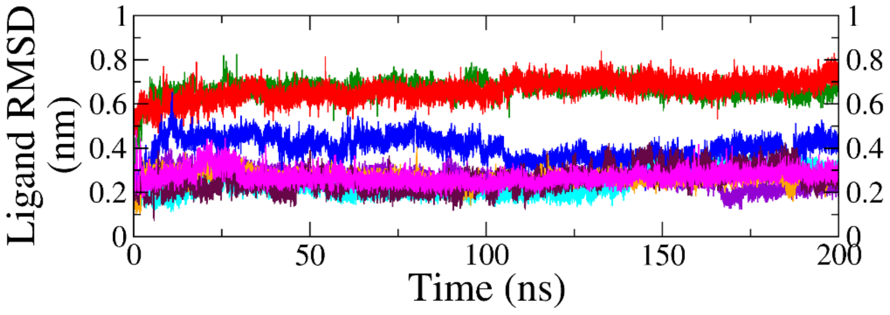

Fig 4. RMSD of top 8 ligands relative to protein backbone obtained from MDS trajectories of different complexes (violet= beta-sitosterol, green= squalene, red= etretinate, cyan= rhoifolin, magenta= swertisin, blue= phloridzin, orange= rhapontin, maroon= diosmetin-7-O-beta-D-glucopyranoside).

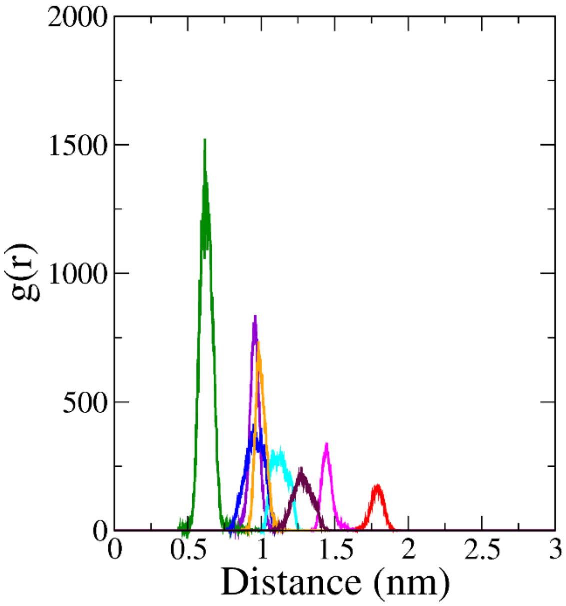

Fig 5. RPDF plot depicting the center of mass between the top 8 ligands and the protein within these complexes extracted from the MDS trajectories; a distinct sharp peak

indicates the ligand's localized positioning; Complex 1 (violet); Complex 2 (green); Complex 3 (red); Complex 4 (cyan); Complex 5 (magenta); Complex 6 (blue); Complex 7 (orange); Complex 8 (maroon).

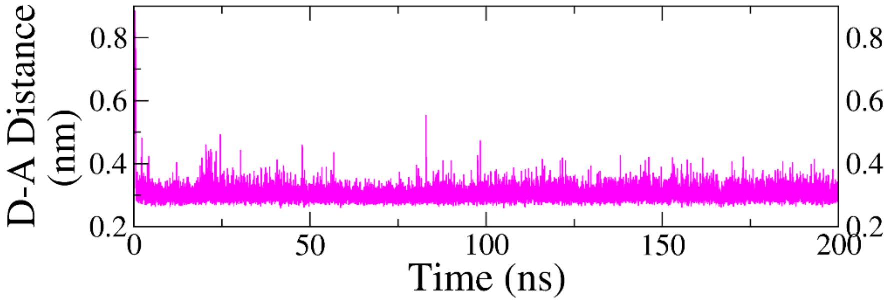

(a)

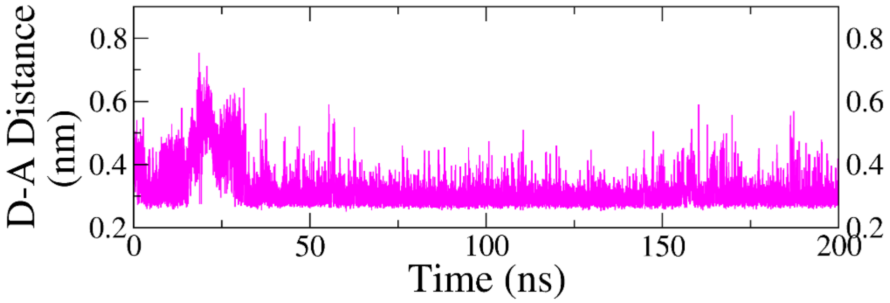

(b)

Fig 6: Changes in D-A atom distances in hydrogen bonds observed during the MDS trajectory for protein-ligand complex with swertisin, involving a) HIS90; b) TYR435 and the oxygen acceptor of the ligand, swertisin.

Please let us know if any problems persist, and we will be happy to assist further.

2. Please discuss the limitations of the computational methodologies in drug discovery. For example please list limitations of Molecular docking and MDS.

Response: The specific limitations of molecular docking have been thoroughly addressed in the Molecular docking calculation subsection of the Computational method section. Additionally, a broader discussion addressing the limitations of molecular docking, molecular dynamics simulations (MDS), and computational methodologies in general has been incorporated into the concluding paragraph of the Results and Discussion section, immediately preceding the Conclusion.

3. How these computational methodologies are better than the bench work and how each predicted target can be biologically tested.

Response: The advantages of computational methodologies over traditional bench work, along with the approaches for biological validation of predicted targets, have now been addressed in the revised manuscript. This discussion can be found in lines 123 to 133 of the Introduction section.

4. Please discuss in detail the validations of the predicted targets.

Response: The biological relevance of the predicted target was assessed through an extensive review of the literature, which confirmed its well-established involvement in the pathogenesis of neurodegenerative disorders. Although direct experimental validation was not performed in this study, the selection of monoamine oxidase B (MAOB) as the key target is strongly supported by previous research demonstrating its critical role in Alzheimer's and Parkinson's diseases, as detailed in the Introduction and Computational method (Target Selection) sections. This provides an indirect yet robust validation of our computational prediction. Nonetheless, we acknowledge

that further experimental studies are required to empirically validate these findings and strengthen the conclusions and we have accordingly recommended such investigations.

5. Explain the docking score

Response: The explanation of the docking score has now been included in the Molecular Docking Calculations subsection of the Computational Method section (lines 191–196).

Reviewer 2:

Most of my comments have been fully addressed, with the exception of:

H-Bond Count and H-bond distribution

6. Include the image for complex 2 in Fig S6, especially as it shows no Hydrogen bonds. (This update still appears to be missing from the revised Supporting Information).

Response: The hydrogen bond count plot for Complex 2 has now been included in Figure S6 of the revised Supporting Information. As correctly noted, Complex 2 does not exhibit any hydrogen bonding throughout the simulation, which is reflected by the flat line in the hydrogen bond count plot **(2)** (Fig S6.). Consequently, a hydrogen bond distribution curve has not been presented for this complex, as no hydrogen bonds were detected during the trajectory.

Reviewer 3:

(No Response)
