# Supplementary material for: Evaluating the inhibitory efficacy of Oxalis phytocompounds on monoamine oxidase B: An integrated approach targeting age related neurodegenerative diseases through molecular docking and dynamics simulations
Source: PLoS One. 2025 Jul 30;20(7):e0329168. doi: 10.1371/journal.pone.0329168 (PMC12309996; doi:10.1371/journal.pone.0329168)
Supplement: Attachment — Submitted filename: review_DO.docx [file pone.0329168.s002.docx]

Congratulations on your manuscript titled; *“Evaluating the Inhibitory Efficacy of Oxalis Phytocompounds on Monoamine Oxidase B: An Integrated Approach Targeting Age Related Neurodegenerative Diseases through Molecular Docking and Dynamics Simulations.”*

To improve its suitability for publication, I will recommend working on the references & Figure citations and other points outlined below.

**Introduction**

1. Wilson et al., 2023 should be cited as number 1 reference and update others appropriately.

**Molecular interactions in different adducts**

2. Cite the complexes structure in the SI: “Notably, hydrophobic interactions were found as the predominant type of interactions in Complex 1, Complex 2, Complex 3, and Complex 5 (see supporting information)”

**Geometrical Analysis of Adducts Conformation**

3. Provide an appropriate citation and reference for; “The adduct is considered more stable if the ligand stays bound to the orthosteric site for a longer duration while maintaining its orientation and location relative to the protein backbone”
4. Specify which complex contains Beta-sitosterol; “**In complex X**, Beta-sitosterol exhibited only minor orientation changes at 50, 100, 150, and 200 ns compared to its position at 1 ns in the adduct”

**Root mean square deviation (RMSD)**

5. Provide an appropriate citation and reference for; “. A higher RMSD suggests greater deviation from the reference conformation over time, while a stable RMSD suggests system equilibration, and persistent fluctuations may imply ongoing structural adjustments”

**H-Bond Count and H-bond distribution**

6. Include the image for complex 2 in Fig S6, especially as it shows no Hydrogen bonds.

**Energetic analysis of protein-ligand adduct formation**

7. Remove word editor box around the text; “Table 2 presents the binding free energy changes of the complexes, calculated from the equilibrated segment of the MDS trajectory.”
8. Remove the text if not applicable“ ∆G_Gas_= Gas phase energy change, ∆G_Sol_ = Electrostatic solvation energy change”
9. Fig S8 image is missing in the supporting information.
10. Remove word editor box around the text; “Additionally, Fig S8 in the supplementary information, provides a detailed frame-by-frame analysis of the binding free energy change (∆G_BFE_) across eight distinct complexes throughout the equilibrated phase. It revealed that the moving average consistently stayed within the negative range”
11. Rewrite the entire paragraph for clarity: “It revealed that the moving average consistently stayed within the negative range for all complexes indicating sustained spontaneity of the adduct formation reaction. All the data pointed towards stable nature of the adducts and the hit candidates are possibly capable of inhibiting the functioning of the receptor MAO-B. The inferences derived from theoretical procedure forms the basis for experimental validation in the drug design and discovery procedural pipeline. “
